# A CW-type zinc finger protein is involved in RES-oxylipin signaling and the response to abiotic stress in *Arabidopsis thaliana*

**DOI:** 10.3389/fpls.2025.1535643

**Published:** 2025-03-12

**Authors:** Manuel Lange, Arthur Korte, Maximilian Fuchs, Agnes Fekete, Claudia Mueller, Barbara Dierich, Jannis Witte, Thomas Dandekar, Martin J. Mueller, Susanne Berger

**Affiliations:** ^1^ Pharmaceutical Biology, Julius-von-Sachs-Institute for Biosciences, University Wuerzburg, Wuerzburg, Germany; ^2^ Botany 2, Julius-von-Sachs-Institute for Biosciences, University Wuerzburg, Wuerzburg, Germany; ^3^ Fraunhofer Institute for Toxicology and Experimental Medicine, Hannover, Germany; ^4^ Chair of Bioinformatics, University Wuerzburg, Wuerzburg, Germany

**Keywords:** RES-oxylipins, stress signaling, zinc finger protein, *Arabidopsis*, oxidative stress, detoxification

## Abstract

**Introduction:**

Oxylipins regulate the response of plants to biotic and abiotic stress factors and the tolerance of unfavorable conditions. While the signaling pathway of jasmonic acid has been intensively studied, little is known about the signal transduction that mediates the responses of reactive electrophile oxylipins such as 12-oxo phytodienoic acid and prostaglandins.

**Methods and results:**

Here, a CW-type zinc finger protein (ZIFI1, At3g62900) was identified as a new signaling factor in a mutant screen. Transcriptome analysis of *Arabidopsis* mutants with a defect in this gene showed that the zinc finger protein is involved in regulating gene expression. Only about half (327 genes) of the about 646 genes induced by the reactive electrophilic oxylipin prostaglandin in the wild type was also up-regulated in the *zifi1* mutant. The differentially expressed genes are enriched in genes related to detoxification and responses to stress factors such as oxidative stress. Therefore, it was tested whether a defect in the zinc finger gene resulted in altered sensitivity to stress factors. The sensitivity to the reactive oxygen species butyl hydroperoxide and to the xenobiotic triiodobenzoic acid was increased in the mutant. In addition, production of reactive oxygen species induced by the bacterial elicitor flg22 was accelerated.

**Discussion:**

The results provide new insights into the factors involved in the signaling of reactive electrophiles and the connection of different stress signaling pathways.

## 1 Introduction

Environmental stress conditions often result in the production of reactive compounds. This might be the consequence of the stress, or the production might be on purpose being part of defense reactions or both. Reactive compounds comprise reactive oxygen species (ROS) as well as organic compounds with reactive structural features (Knieper et al., 2023). There are several reports that compounds of both groups accumulate upon stress factors and function in stress signaling (Almeras et al., 2003; Mittler et al., 2022). Both groups are connected in that ROS can contribute to the production of reactive compounds. For instance, lipid peroxidation by ROS leads to the formation of oxidized lipids such as phytoprostanes and malondialdehyde (Farmer and Mueller, 2013). In addition, the accumulation of reactive compounds might lead to enhanced levels of ROS.

Reactive electrophile species (RES) are characterized by their tendency to react with nucleophilic groups. The electrophilic reactivity is consists of diverse structural features. One group consists of isothiocyanates which are defense compounds, prominently found in the family of Brassicaceae (Burow and Halkier, 2017). These compounds are present as non-reactive glucosinolates which are converted to reactive isothiocyanates by myrosinases after tissue damage.

Another important group of RES comprises RES-oxylipins which contain an α,ß-unsaturated carbonyl structure (Knieper et al., 2023). Examples of RES-oxylipins in plants are 12-oxo-phytodienoic acid (OPDA), phytoprostanes, and hexenal. Several RES-oxylipins accumulate in response to stress and are biologically active (Croft et al., 1993; Almeras et al., 2003; Grun et al., 2007). Among the RES-oxylipins, OPDA is the most studied compound. OPDA is an enzymatically formed cyclopentenone oxylipin that can be further converted to jasmonic acid (JA), and the application of OPDA will elicit CORONATINE INSENSITIVE 1 (COI1)-mediated responses via JA-isoleucine. In addition, OPDA has signaling functions independent of its conversion to JA. In *Arabidopsis*, OPDA is involved in the regulation of stomatal closure in response to drought and hypoxia (Savchenko et al., 2014). OPDA is also involved in defense against the necrotrophic fungi *Alternaria brassicicola* and *Botrytis cinerea* (Raacke et al., 2006). Also, products of the 9- and 13-lipoxygenase pathway such as 9- and 13-keto-octadecadienoic/keto-octadecatrienoic acids can regulate stomatal closure (Montillet et al., 2013). Furthermore, non-enzymatically formed RES-oxylipins and fragmented oxylipins such as A_1_-, B_1_-, and dJ_1_-phytoprostanes; malondialdehyde; and hexenal increase upon stress conditions and exhibit biological activity (Croft et al., 1993; Almeras et al., 2003; Grun et al., 2007). Regulation of gene expression has been reported for several RES. A considerable overlap of genes responsive to several RES-oxylipins comprises genes related to detoxification and heat stress (Mueller et al., 2008; Yamauchi et al., 2015). Particularly, heat-stress-related genes are also induced by the isothiocyanate sulforaphane and the ROS H_2_O_2_ (Ferber et al., 2020).

Although RES-oxylipins are accepted to function as signaling compounds, the signal transduction mechanisms mediating the biological activities of RES-oxylipins are still only partly known. The perception mechanism for RES-oxylipins is not clear yet. It has been shown that most responses are independent of the JA-isoleucine-JAZ-coreceptor COI1. An OPDA-binding protein, cyclophilin 20-3, has been reported which is involved in mediating part of OPDA-induced effects (Park et al., 2013). Rather downstream in the signaling pathway, TGA transcription factors have been shown to be important for the induction of genes in response to OPDA, A_1_-phytoprostanes, and prostaglandin A_1_ (Mueller et al., 2008; Stotz et al., 2013). However, only 30% of gene induction by OPDA was dependent on TGA factors. To identify new factors of RES-oxylipin signaling, we performed a screen for mutants that are less responsive to RES-oxylipins.

Because of their reactivity, RES at higher concentrations have toxic effects on cells. A typical reaction is the formation of covalent adducts with nucleophiles such as thiol and amino groups in peptides and proteins (Mueller and Berger, 2009). To avoid unintended reactions of RES, organisms have developed mechanisms to metabolize RES to less reactive compounds. These detoxification mechanisms include conjugation with glucose or glutathione. The covalent bond to glutathione can be formed non-enzymatically or enzymatically which is catalyzed by glutathione-S-transferases (GSTs). Members of the GST family have in general a broad substrate specificity. Expression of a high proportion of GSTs is upregulated by stresses and RES such as isothiocyanates, OPDA, A_1_, and phytoprostanes (Mueller et al., 2008; Hara et al., 2010).

This work reports the identification of the CW-type zinc finger protein ZIFI1 which is involved in the regulation of gene expression in response to RES-oxylipins. A defect in this gene resulted in lower expression of genes related to different abiotic stresses, particularly detoxification. The sensitivity of a *zifi1* mutant to oxidative stress and the xenobiotic 2,3,5-triiodobenzoic acid (TIBA) was increased.

## 2 Materials and methods

### 2.1 Plant cultivation and treatment with RES

The T-DNA insertion lines SALK_064820 (for At3g62900, *ZIFI1* gene) and SALK_130710 (for At3g61340, *F-box* gene) were received from the stock center, and the presence of the insertion was verified by PCR. Col-0 was used as the corresponding wild type. The GST6::LUC line was used as the control line for the *nr1* mutant (see also **Supplementary Figure 1**). Seeds were sterilized with chlorine gas. For gene expression analysis upon prostaglandin A_1_ (PGA) and sulforaphane (both Cayman Chemical, Ann Arbor, USA) treatments, sterilized seeds were cultivated in liquid Murashige and Skoog medium with 3% sucrose under short day conditions (9 h light, 160 µE, 22°C) on an orbital shaker. Two days of incubation in the refrigerator preceded the exposure to light. After 7 days, the medium was exchanged. Ten-day-old seedlings were treated by exchanging the medium to a 0.5-mL medium containing 75 µM of PGA, 75 µM of OPDA, 75 µM of sulforaphane, or 0.5% MeOH as mock.

### 2.2 Growth on TIBA

Sterilized seeds were sown on agar plates with Murashige and Skoog medium containing 3% sucrose and 0.1 mM of 2,3,5-triiodobenzoic acid. After 2 days in the refrigerator, plates were placed horizontally under short day conditions (9 h light, 160 µE, 22°C). Germination and growth were scored after 14 days in the light.

### 2.3 Ion leakage

Plants were grown for 6 weeks in pricking soil (SP pikier, Einheitserdewerkverband, Sinntal, Germany) under short day conditions (10 h light, 160 µE). Leaf discs of 5 mm were punched and floated overnight on water at 22°C with slow agitation. Leaf discs were placed in a 24-well plate with 1 mL of water and five discs per well. The water was exchanged for the treatment solution containing 1 mM of tert-butyl hydroperoxide, and controls were treated with water. Samples for gene expression were harvested after 1.5 h. Ion leakage was determined at different time points with a conductivity device (Horiba, Lelystad, Netherlands). After 27 h, leaf discs were incubated at 100°C to kill the cells, and maximal ion leakage was measured after cooling down. Ion leakage was calculated as the percentage of maximal ion leakage.

### 2.4 ROS measurements

Plants were grown for 36 days in pricking soil under short day conditions (10 h light, 160 µE). Leaf discs of 5 mm were punched and floated overnight on water at 22°C with slow agitation. Leaf discs were placed in a 96-well plate (one disc per well) with the lower side down floating on 100 µL of water and incubated for 3 h in the dark. The water was exchanged for 50 µL of measuring solution containing 0.006 mg/mL of luminol, 2% DMSO, and 0.006 mg/L of horse radish peroxidase. Another 50 µL of measuring solution containing 100 nM of flg22 was added immediately before the start. Measurement was performed with the Clariostar plus (BMG Labtech, Ortenberg, Germany) in 2-min intervals with a measuring time of 0.8 s/well.

### 2.5 Mutant screen

Seeds of the GST6::LUC line were mutagenized with ethyl methanesulfonate (Sigma, Saint Louis, USA). Seeds (100 mg) were incubated for 16 h in 0.1 M of sodium phosphate buffer pH 5 at 4°C. Ethyl methanesulfonate was added to a concentration of 0.4%, and incubation was performed for 8 h on a shaker at room temperature. Seeds were washed twice with 0.1 M of sodium thiosulfate and two times with distilled water. Plants were grown in soil, and after self-pollination, seeds of single M1 plants were collected separately. These seeds of the M2 generation were used for screening to be able to detect recessively inherited mutant phenotypes. M2 seeds of 3,257 independent M1 lines were grown in 96-well plates in liquid medium (one plant per well, eight plants per line) and screened for lower induction of luciferase activity after treatment with 75 µM of PGA in comparison to the non-mutagenized GST6::LUC line. Putative less-responsive mutants were selected and retested in the next generation. Backcrosses to the non-mutagenized GST6::LUC line were performed, and the next two generations were tested for segregation of the phenotype of less luciferase activity after PGA treatment. For measurement of luciferase activity, 50 µL of a 1-mM luciferin solution containing 0.01% Triton X-100 was added to each well. After 10 min of exposure to darkness, light emission was collected for 10 min with a CCD camera (Hamamatsu C4742-98; Hamamatsu Photonics). The Imaging Software Hokawo 2.1 (Hamamatsu Photonics) was used for image acquisition. For quantitative analysis, the collection of light emission was for 15 min and the mean of mock controls was subtracted.

### 2.6 Mapping of *nr1*

To establish a mapping population, the backcross of the *nr1* mutant to the non-mutagenized GST6::LUC control line was used. F1 plants were grown and seeds of the F2 generation were harvested after self-pollination. Plants of the F2 were tested for the mutant phenotype (less luciferase activity after PGA treatment). A population of 50 F2 plants with mutant phenotypes was selected and the mutant phenotype was confirmed in the F3 generation of these plants. DNA of the 50 F2 plants was pooled, sequenced, and compared to the Col-0 wild type and the GST6::LUC control line. To identify genomic regions that correlate with the observed phenotype, bulked segregant analysis was performed using SHOREmap v3.5 (Schneeberger et al., 2009). This analysis revealed that the causative mutation is presumably located at the end region of chromosome 3 (**Supplementary Figure 2**). Detailed analysis could identify a mutation in the *nr1* gene that leads to an exchange of a G to an A (Chr 3, position 23252228) in the *nr1* sequence which results in a premature stop codon.

### 2.7 Generation of complementation lines

The complete coding sequence of *ZIFI1* (At3g62900.1) of 4,212 bp with additional attb recombination sites for gateway cloning of 61 bp was synthesized and inserted in the vector pUC53 (Genewitz, Leipzig, Germany). By BP reaction, the CDS was transferred to the entry vector pDONR201 and by LR reaction to the destination vector pB2GW7. *Agrobacterium tumefaciens* strain GV3101 was transformed with pB2GW7Zifi. A floral dip of *nr1* mutant plants with pB2GW7Zifi containing *Agrobacterium* suspension was performed as described in Clough and Bent (1998). Plants containing the transgene were selected by Basta resistance and expression of the *ZIFI1* gene was analyzed by RT-qPCR.

### 2.8 RNA isolation and quantitative RT-PCR

Approximately 100 mg of plant material was ground in a mixer mill at 21 Hz. Total RNA was extracted using NucleoZOL (Machery and Nagel, Dueren, Germany) according to the manufacturer’s protocol. RNA concentration was determined spectrophotometrically. The remaining DNA was removed using RNase-free DNase I (Fermentas, St. Leon-Rot, Germany) according to the manufacturer’s protocol. First-strand cDNA and real-time PCR were performed as described previously (Szyroki et al., 2001) using SYBR-Green Capillary Mix (Thermo Fisher Scientific, Hamburg, Germany) and a CFX 96™ Real-Time System C1000™ Thermal Cycler (Bio-Rad, CA, USA). The primers used (Merck and TIB MOLBIOL, Berlin, Germany) are given in **Supplementary Table 1**.

### 2.9 Transcriptome analysis

RNA was extracted from approximately 100 mg of plant material using NucleoZOL (Machery and Nagel, Dueren, Germany) as described above. RNA was purified using the RNeasy kit (Qiagen, Hilden, Germany). RNA concentration was determined spectrophotometrically. RNA integrity was confirmed using an Agilent RNA 6000 Nano Chip on an Agilent 2100 BioAnalyzer (vB.02.03 BSI307). Labeling and hybridization were performed by the array facility of the University of Erlangen. Labeling and preparation of samples for hybridization were conducted as described in the one-color microarray-based gene expression analysis protocol provided by Agilent including the one-color RNA spike-in kit (v5.0.1, 2006; Agilent Technologies, Santa Clara). Slides were scanned on the Agilent Microarray Scanner with extended dynamic range (XDR) at high resolution (5 μm). Data sets were extracted by feature extraction software package (v11.5.1.1/Agilent Technologies) using a standard protocol (GE1_1105_Oct12). Three independent, biological replicates were analyzed.

Raw microarray data were imported and processed using the read.maimages function in R. Initial quality control included the inspection of raw data for quality metrics and the filtering of low-quality probes. Background correction and normalization were carried out using the limma package, with quantile normalization applied to ensure consistency across arrays. Differential gene expression analysis was performed using limma. An adjusted *p*-value threshold of 0.05, corrected for multiple testing using the Benjamini–Hochberg method, was applied to identify differentially expressed genes. Only genes with a log fold change greater than 1 or less than −1 were considered significant. For exploratory data analysis, principal component analysis (PCA) was conducted using the prcomp function. The results were visualized with ggplot2 to assess sample clustering and identify potential outliers. Functional overrepresentation analysis of differentially expressed genes (DEGs) was performed using the gprofiler2 package, with probe annotations based on GPL9020.

### 2.10 Analysis of glutathione conjugates

Thirty to 80 mg of plant material was used for each sample. Extraction and measurement were performed as described in Ferber et al. (2020). A 10-µL extraction solution (methanol:water:formic acid 9:1:0.1) containing 8.5 mM of methane thiosulfonate and 10 nmol of glutathione ethylester as internal standard was added per mg seedling material. After incubation for 2 min at 80°C, the samples were homogenized using a ball mill and centrifuged for 10 min at 10,000*g* and 4°C. The supernatant was transferred to a new tube, cleared by centrifugation, and used for analysis with an Acquity UPLC coupled with a quadrupole/time-of-flight mass spectrometer (Synapt G2 HDMS, Waters, Milford, MA, United States). Separation was performed using a BEH C18 column (2.1 × 100 mm, 1.7 µm, Waters) with a precolumn. The column temperature was 40°C. Eluent A was 0.1% formic acid in water, and eluent B was 0.08% acetonitrile in water. Eluent B was added in a gradient of 0%–60% over 5 min. The flow rate was 0.3 mL/min. Mass spectroscopy was performed in a mass range of 50–1,200 Da using negative electrospray ionization.

### 2.11 Statistics

For the statistical analysis of the differences between treatment and control or between the mutant and control lines, the Student’s *t*-test or two-way ANOVA with Tukey’s HSD test was used as specified in the legends.

## 3 Results

### 3.1 A zinc finger protein is involved in RES-oxylipin signaling

**Figure 1:**
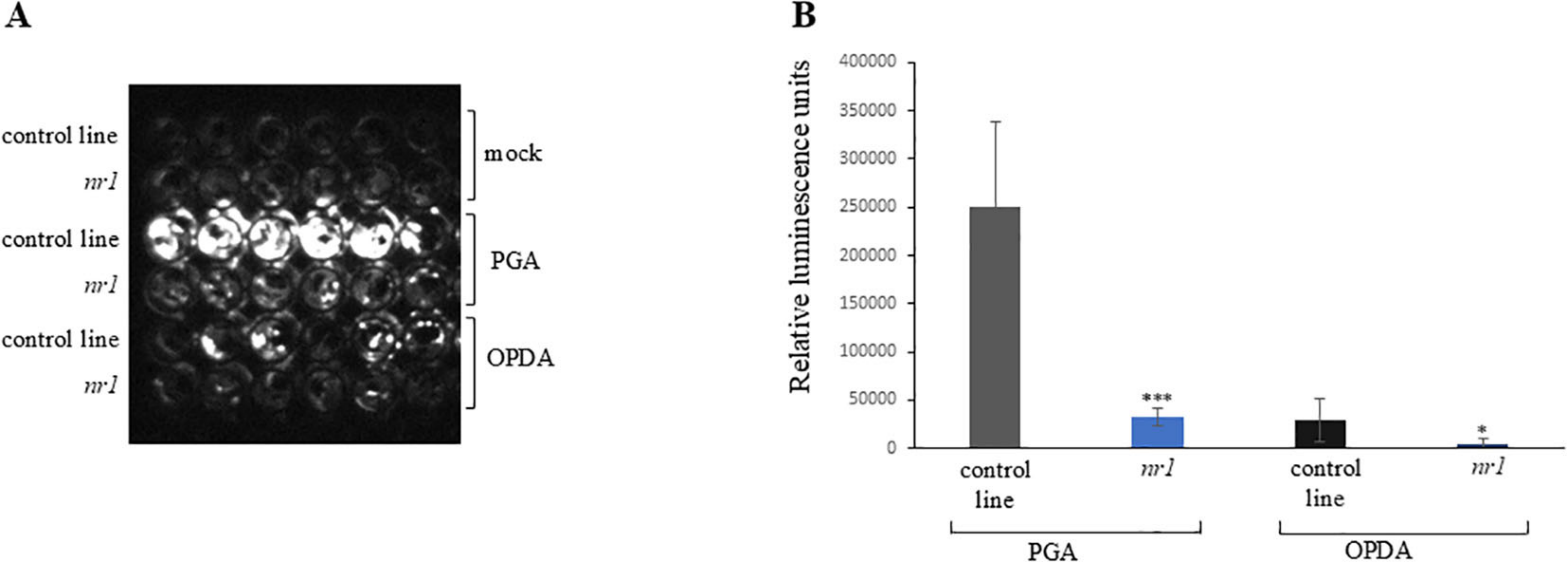
Luciferase activity of the *nr1* mutant after oxylipin treatment. Ten-day-old seedlings were treated with mock (MeOH), 75 µM of PGA, or 75 µM of OPDA. The control line is the non-mutagenized GST6::LUC line. **(A)** Luminescence was recorded with a CCD camera 7 h after treatment. Shown are six replicates of each genotype and treatment. **(B)** Luminescence of each well was determined 7 h after treatment. Shown is the mean of six replicates of each genotype and treatment ± SD. Stars indicate significant differences between *nr1* and the control line (*p < 0.05; ***p < 0.001). The experiment was repeated two times with similar results.

In order to identify new components of RES-oxylipin signaling pathways, a non-biased forward genetic approach was pursued. The GST6::LUC reporter line (Chen and Singh, 1999) was mutagenized and a mutant population was screened for altered luciferase activity upon RES-oxylipin treatment. The *GST6* gene encodes a GST which can detoxify RES. This enzyme can conjugate OPDA and A_1_-phytoprostanes with glutathione which probably contributes to the inactivation of these RES-oxylipins (Mueller et al., 2008). Prostaglandin A_1_ (PGA) was used for the mutant screen as a model RES-oxylipin. PGA is a signaling compound in animals and is structurally related to A_1_-phytoprostanes and OPDA. PGA treatment results in a similar and even stronger induction of RES-oxylipin responsive genes such as *GST6*, *OPR1*, *CYP81D11*, and *GST25* (Stotz et al., 2013). PGA has the advantage that—in contrast to OPDA—it cannot be converted to JA. A total of 3,257 independent M1 lines were screened for lower induction of luciferase activity after treatment with 75 µM of PGA in comparison to the non-mutagenized GST6::LUC line. The mutant *nr1* (non-responsive1) showed pronounced lower luciferase activity after PGA treatment compared to the control line (**Figures 1A, B**). This phenotype was recessively inherited. To investigate, whether also the response to the endogenous RES-oxylipin OPDA is altered, induction of luciferase activity by OPDA was tested. The *nr1* mutant showed significantly less induction of luciferase activity by OPDA compared to the control line (**Figures 1A, B**).

Bulked segregant analysis revealed a region at the end of chromosome 3 that showed significant allele frequency changes between wild-type and mutant plants (**Supplementary Figure 2**). This analysis indicated that the causative mutation is located in this region, which contains two possible candidate genes with non-synonymous base changes within the coding sequence. Both SNPs show an allele frequency for the respective SNP of 1 in the pooled mutant lines. The two candidate genes encode a CW-type zinc finger protein (At3g62900, ZIFI1) and an F-box protein (At3g61340), respectively.

**Figure 2:**
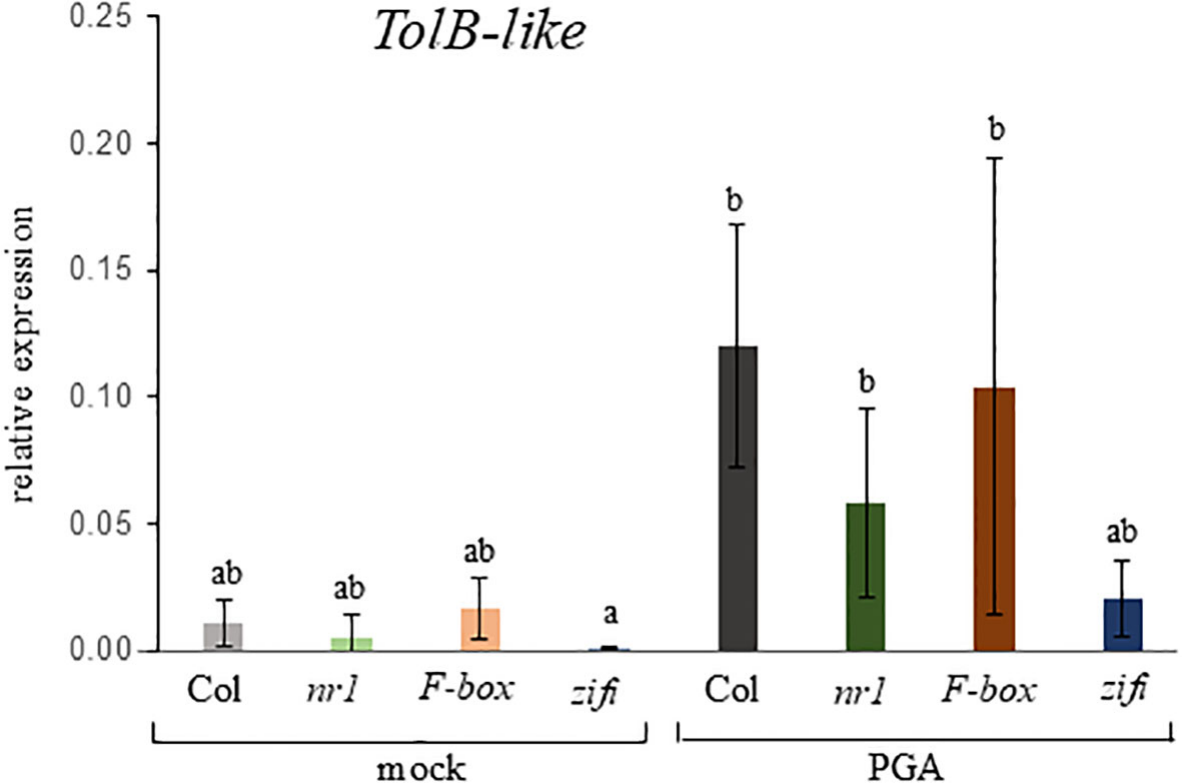
Expression of the RES-oxylipin-responsive gene *TOLB-like* in different mutant lines. Seedlings of wild type Col (gray bars), *nr1* (green bars), and the T-DNA-insertion lines F-box (red bars) and *zifi1* (blue bars) were treated with mock or 75 µM of PGA for 4 h. Expression is relative to *SAND*. Shown is the mean of four biological replicates ± SD. Different letters above the bars indicate statistically significant differences (two-way analysis of variance followed by Tukey’s HSD test, *p* < 0.05). The experiment was repeated with similar results.

To find out which of these mutations is responsible for the lower response to PGA, expression of the RES-oxylipin responsive gene *TolB-related* was analyzed in separate T-DNA insertion lines with a defect in either one or the other of the two candidate genes. *TolB-like* expression after PGA treatment was lower in seedlings of *nr1* and the line defective in the *ZIFI1* gene, while in the line with a defect in the F-Box gene expression was similar to the wild type (**Figure 2**). In a second approach, the wild type coding sequence of the *ZIFI1* gene was expressed in the *nr1* background. Expression of the *ZIFI1* gene showed in different lines at least partial complementation of PGA-induced luciferase activity (**Supplementary Figure 3**).

Based on the results of both approaches, it was concluded that the mutation in the *ZIFI1* gene is causative for the lower sensitivity to RES-oxylipins.

### 3.2 The zinc finger gene shows little regulation on a transcriptional level

So far, no functional roles have been assigned to this protein. Often, the function of proteins is mirrored by a transcriptional regulation related to the function (Zimmermann et al., 2004). Based on the information in Genevestigator and the eFP Browser, expression of *ZIFI1* was at medium to high levels in different organs and at different developmental stages (**Supplementary Figures 4A, B**). Different treatments resulted only in little up- or downregulation of *ZIFI1* expression (**Supplementary Figure 4C**). In summary, the expression profile of the *ZIFI1* gene did not give strong indications for the biological function of this protein.

### 3.3 Genes related to stress responses show altered expression in the zinc finger mutant

The zinc finger is a DNA-binding motif and zinc finger proteins are often transcriptional regulators. Therefore, transcriptome analysis was employed to reveal the function of this protein. To avoid the effects of the mutations in *nr1* which are close to the *ZIFI1* gene and do not segregate (e.g., in the F-Box gene), for further studies, the T-DNA insertion line (*zifi1*) with a defect in the expression of *ZIFI1* was preferentially used. The transcript level of the *ZIFI1* gene was in *nr1* only non-significantly lower (**Supplementary Figure 5**). The mutation in *nr1* results in a stop codon at position 1737 of the coding sequence or after 579 amino acids of 1,403 amino acids, respectively. In the SALK line, the T-DNA insertion is located in the fifth exon. *ZIFI1* transcript levels in this line are only slightly above the detection limit (**Supplementary Figure 5**). The mutant showed overall normal development. However, we noticed that hypocotyls of *zifi1* seedlings were slightly shorter than the wild type, and the fresh weight of soil-grown plants was lower in most experiments (**Supplementary Figure 6**).

**Figure 3:**
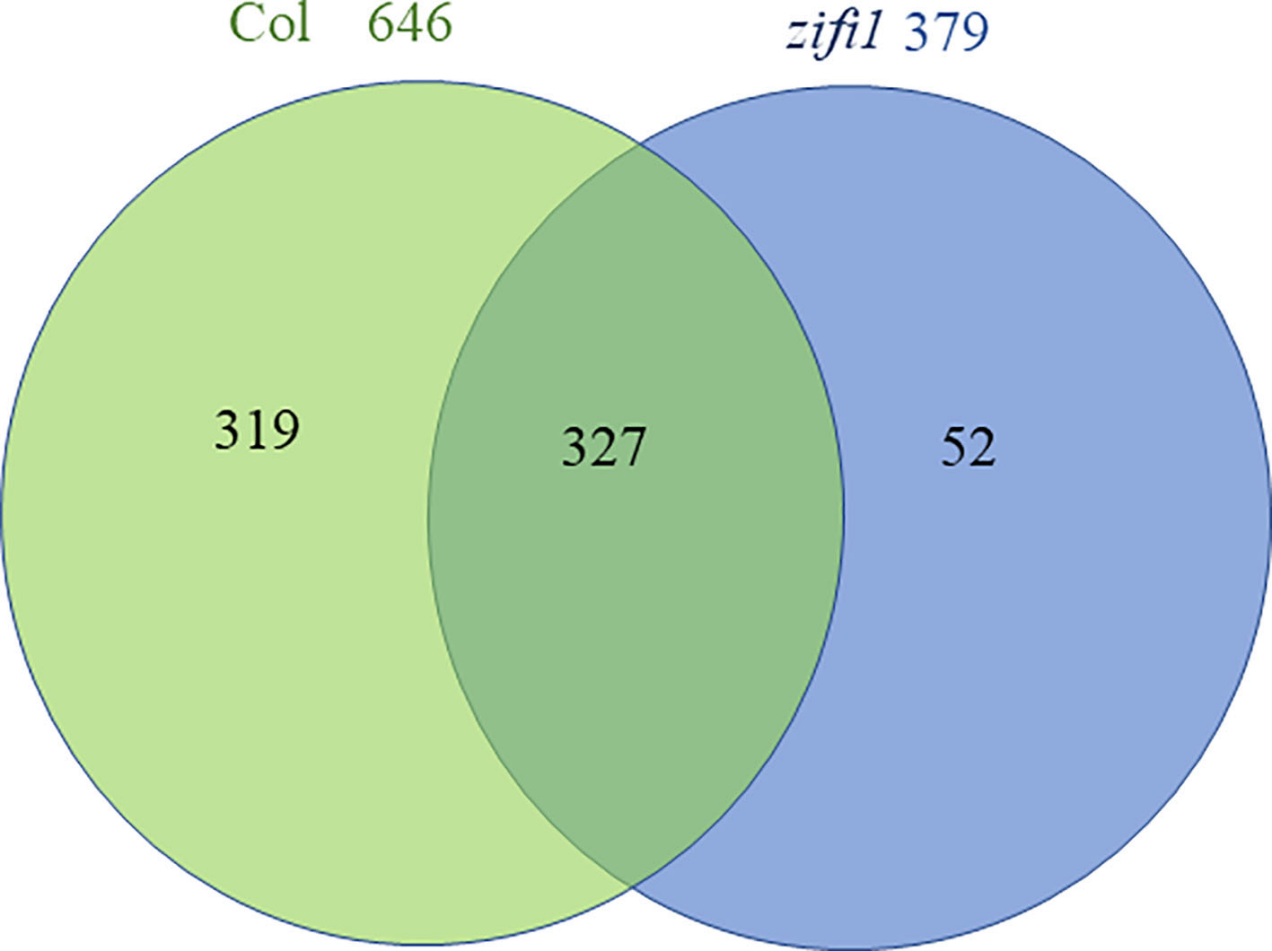
Venn diagram comparing genes upregulated by PGA in wild-type and *zifi1* plants. Expression was analyzed in wild-type (Col) and *zifi1* plants at 4 h after treatment with 75 μM PGA of PGA in comparison with mock-treated controls. Three biological replicates were used for each genotype and treatment.

Transcriptome analysis of mock-treated and PGA-treated seedlings of wild type, *nr1*, and the *zifi1* mutant was performed. In mock-treated *zifi1* seedlings, 331 genes were more than a factor 2 lower and 154 genes were more than twofold higher expressed than in the wild type, respectively (**Supplementary Table 2**). Upon PGA treatment, substantially more genes showed differential regulation. There were 754 genes that showed lower expression and 335 genes that showed higher expression in *zifi1* compared to Col-0, respectively (**Supplementary Table 3**). There was a substantial overlap in the differentially regulated genes in the *nr1* and *zifi1* mutants (**Supplementary Table 4**), and differences between the mutants might be due to additional mutations in *nr1*. The expression of 646 genes was induced by PGA treatment in the wild type, while only approximately half of these genes (327 genes) were also induced in the mutant (**Figure 3**; **Supplementary Table 5**). Only 52 genes showed induction in the mutant but not in the wild type. As expected, a substantial proportion of the genes induced by PGA in the wild type but not in *zifi1* is also responsive to phytoprostanes (49%) and OPDA (33%) in the wild type. This supports that the ZIFI1 protein is involved in regulating gene expression in response to RES-oxylipins.

To elucidate the pathways in which ZIFI1 is involved, gene ontology annotations for the genes showing lower expression in *zifi1* after PGA treatment were evaluated. Remarkably, there was a strong enrichment of genes related to responses to stress (**Table 1**; **Supplementary Table 3**). Especially, responses to abiotic stress such as hypoxia, oxidative stress, and water deprivation were overrepresented. Strikingly, also genes related to detoxification encoding UDP-glycosyltransferases, GSTs, and cytochrome-P450 enzymes were abundant (**Table 1**). Based on these data, phenotypes of the *zifi1* mutant related to detoxification and ROS were investigated.

**Table 1:** Enrichment of selected GO terms in the categories biological process and molecular function of genes with significantly lower expression in *zifi1* compared to Col after PGA treatment.

Term_name	Term_id	Adjusted_p_value	Intersection_size	Term_size
Biological process				
Response to stress	GO:0006950	1.06004E−30	224	3,906
Response to biotic stimulus	GO:0009607	4.84505E−27	121	1,482
Response to bacterium	GO:0009617	1.42503E−16	57	534
Response to fungus	GO:0009620	1.02697E−07	48	650
Response to abiotic stimulus	GO:0009628	9.71036E−20	138	2,214
Response to hypoxia	GO:0001666	7.3376E−20	44	265
Response to oxidative stress	GO:0006979	8.00207E−16	52	465
Response to water deprivation	GO:0009414	1.36628E−07	36	402
Response to abscisic acid	GO:0009737	9.19093E−07	44	603
Response to lipid	GO:0033993	1.25161E−06	59	966
Response to temperature stimulus	GO:0009266	4.0553E−06	44	633
Response to osmotic stress	GO:0006970	1.75604E−05	39	549
Detoxification	GO:0098754	5.14659E−05	24	252
Response to salt stress	GO:0009651	9.46059E−05	34	470
Molecular function				
UDP-glycosyltransferase activity	GO:0008194	3.47252E−07	29	289
Monooxygenase activity	GO:0004497	1.84822E−06	30	330
Glutathione transferase activity	GO:0004364	3.6516E−05	12	64

The complete analysis of GO terms can be found in **Supplementary Table 3**.

### 3.4 Is the zinc finger protein involved in the regulation of detoxification processes?

**Figure 4:**
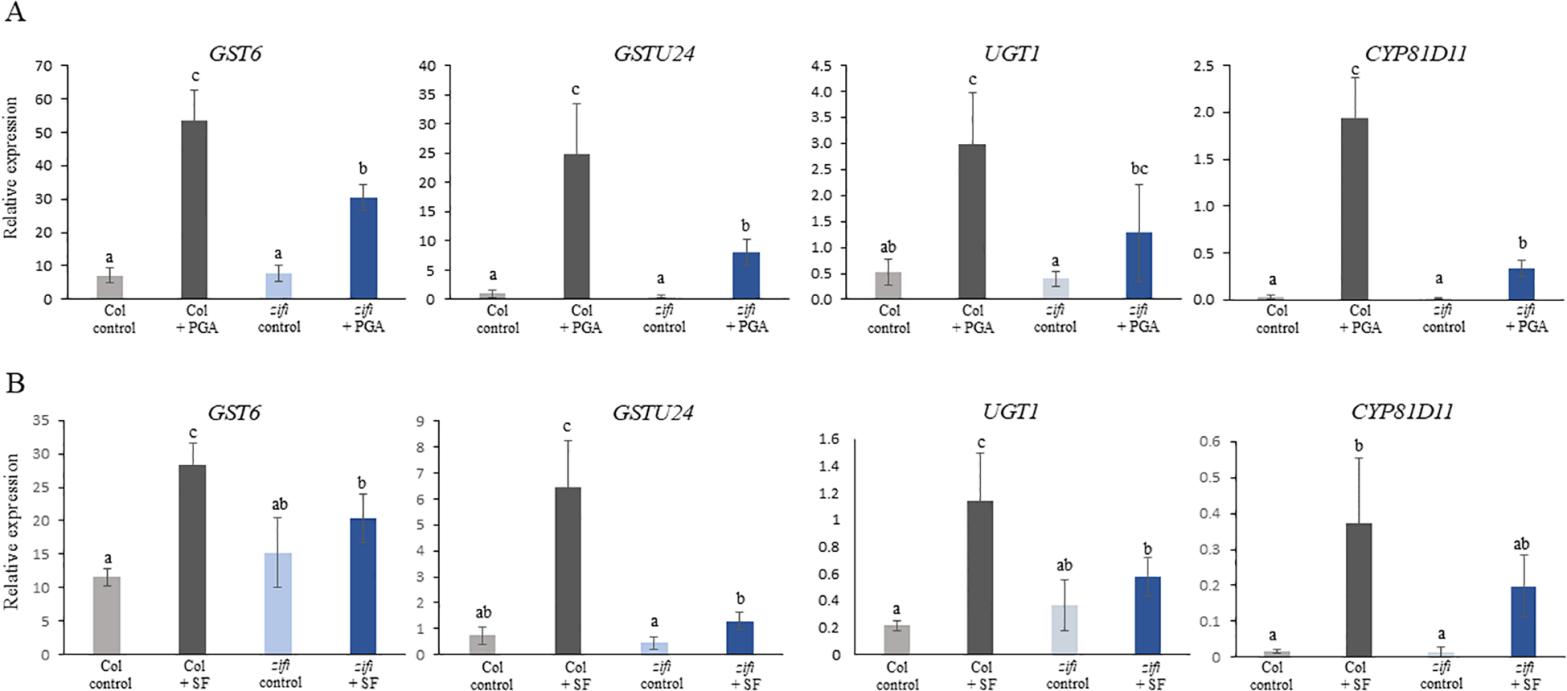
Expression of genes related to detoxification in response to treatment with PGA and SF. RT-qPCR analysis of two GSTs, a glucosyltransferase and a cytochrome P450 enzyme. Seedlings of wild-type Col (gray bars) and *zifi1* (blue bars) were treated with mock (lighter color) or **(A)** 75 µM of PGA (darker color) or **(B)** 75 µM of SF (darker color), respectively, for 4 h. Expression is relative to *SAND*. Shown is the mean of at least four biological replicates ± SD. Different letters above the bars indicate statistically significant differences (two-way analysis of variance followed by Tukey’s HSD test, *p* < 0.05) The experiment was repeated with similar results.

A remarkable number of genes related to detoxification was induced by PGA in the wild type and was not induced or less induced in the *zifi1* mutant. This comprises particularly GSTs and UDP-glucosyltransferases. Comparison of induction based on the transcriptome data is listed in **Table 2**, and expression analysis of selected genes by RT-qPCR is shown in **Figure 4**. Of the 14 GSTs induced by PGA in the wild type, the expression of 13 genes was not upregulated by PGA in *zifi1* or induced to a lower extent. Additional seven GSTs that are not PGA-responsive show lower expression in *zifi1* (**Supplementary Table 3**). Eighteen glycosyltransferases were induced by PGA in the wild type. Except for two genes, these glycosyltransferases showed no or lower induction by PGA in *zifi1*. Similarly, the six PGA-induced ABC transporters exhibited less upregulation by PGA in *zifi1* (**Table 2**).

**Table 2:** PGA-inducible detoxification genes differentially expressed in *zifi1* compared to Col based on transcriptome data from **Supplementary Table 3**.

Gene symbol	logFC PGA vs. control	
	Col	*zifi1*
PGA-responsive GSTs		
ATGSTF8	3.02	2.11
ATGSTU1	1.97	1.81
ATGSTU11	3.55	1.82
ATGSTU16	1.19	0.77
ATGSTU19	2.66	1.75
ATGSTU2	2.55	2.11
ATGSTU22	3.07	2.32
ATGSTU24	5.50	5.39
ATGSTU25	4.61	4.08
ATGSTU4	1.72	0.88
ATGSTU5	1.47	0.71
ATGSTU7	3.42	2.57
ATGSTU8	1.70	0.98
PGA-responsive UGTs		
AT2G22590	2.30	2.03
GT/UGT74F2	1.34	−0.53
GT72B1	1.35	0.57
AT1G05680	4.13	4.45
AT5G03490	1.58	1.11
AT2G30140	1.23	0.72
AT3G46660	1.16	0.19
AT1G73880	1.16	0.68
AT2G36780	1.00	0.14
UGE5	1.43	1.08
UGT1	4.42	1.84
UGT73B1	1.89	0.88
UGT73B2	3.94	3.04
UGT73B3	4.33	3.42
UGT73B4	4.44	3.32
UGT73B5	3.85	2.71
UGT84A1	1.15	0.99
UGT84A2	1.27	1.29
PGA-responsive ABC transporter		
AT3G53510	2.04	1.30
AT5G13580	1.94	1.68
AT5G19410	1.91	1.20
AT3G28345	1.87	0.84
AT1G53270	1.65	0.88
AT3G28345	1.41	0.81

As mentioned above, glucosinolates are RES compounds present in *Arabidopsis*. Since there is a substantial overlap of genes induced by PGA and sulforaphane (Ferber et al., 2020), it was analyzed whether also induction of detoxification genes by sulforaphane was affected in the mutant. Expression of *GST6*, *GST24*, *UGT1*, and *CYP81D11* was in *zifi1* also lower after sulforaphane treatment compared to the wild type (**Figure 4B**). This indicates that besides the response to oxylipins, also the response to additional RES compounds is altered in the mutant.

**Figure 5:**
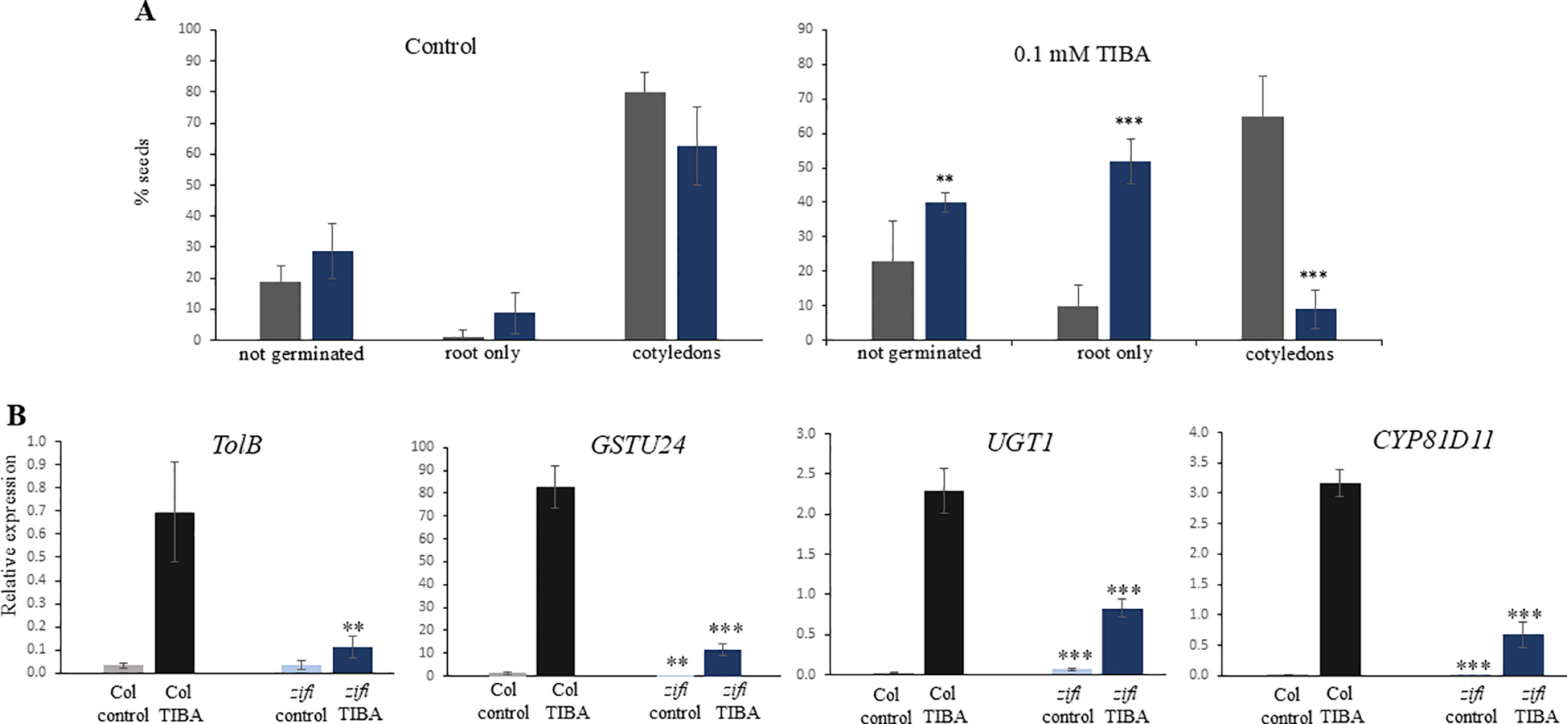
Effect of TIBA on germination, growth, and gene expression. **(A)** Seeds of wild-type Col (gray bars) and *zifi1* (blue bars) were cultivated on plates with 0.1 mM of TIBA (right) or control plates (left). Seedlings that developed only the root or which had developed cotyledons were scored after 14 days. Shown is the mean of 80 seeds for control and 120 seeds for TIBA ± SD. Asterisks indicate significant differences between the wild-type Col and *zifi1* (**p < 0.01; ***p < 0.001). The experiment was repeated with similar results. **(B)** Expression in response to TIBA. Seedlings of wild-type Col (gray bars) and *zifi1* (blue bars) were treated with mock or 0.1 mM of TIBA for 4 h. Expression is relative to *SAND*. Shown is the mean of four biological replicates for *TolB* and *CYP81D11* TIBA and five biological replicates for all other genes and treatments ± SD. Asterisks indicate significant differences between the wild-type Col and *zifi1* (**p < 0.01; ***p < 0.001). The experiment was repeated with similar results.

TIBA is a xenobiotic compound inducing a similar set of genes as the endogenous RES-oxylipins A_1_-phytoprostanes. This comprises especially genes related to the metabolism of xenobiotics. Since the expression of detoxification-related genes is lower in *zifi1*, the sensitivity of this mutant to the xenobiotic TIBA was investigated. Germination and development of *zifi1* on 0.1 mM of TIBA was significantly impaired compared to the wild type (**Figure 5A**). There were 40% of *zifi1* seeds and 23% of wild-type seeds that did not germinate. After 14 days, more than half of the germinated *zifi1* seeds had developed only the root and only 9% had developed cotyledons, while 69% of the wild-type seeds developed cotyledons. Also, gene induction by TIBA was altered in *zifi1*. *TOLB*-related, *GST24*, *CYP81D11*, and *UGT1* were strongly induced by TIBA. Also, in *zifi1*, these genes showed upregulation by TIBA, but the expression levels were significantly lower compared to the wild type (**Figure 5B**).

In summary, this indicates that ZIFI1 is involved in regulating the response to xenobiotics and RES.

### 3.5 ZIFI1 is involved in ROS production and the response to oxidative stress

Since genes related to oxidative stress responses were overrepresented within the genes differentially expressed in wild type and *zifi1*, it was investigated whether the generation of ROS and the tolerance of the mutant to oxidative stress were altered.

**Figure 6:**
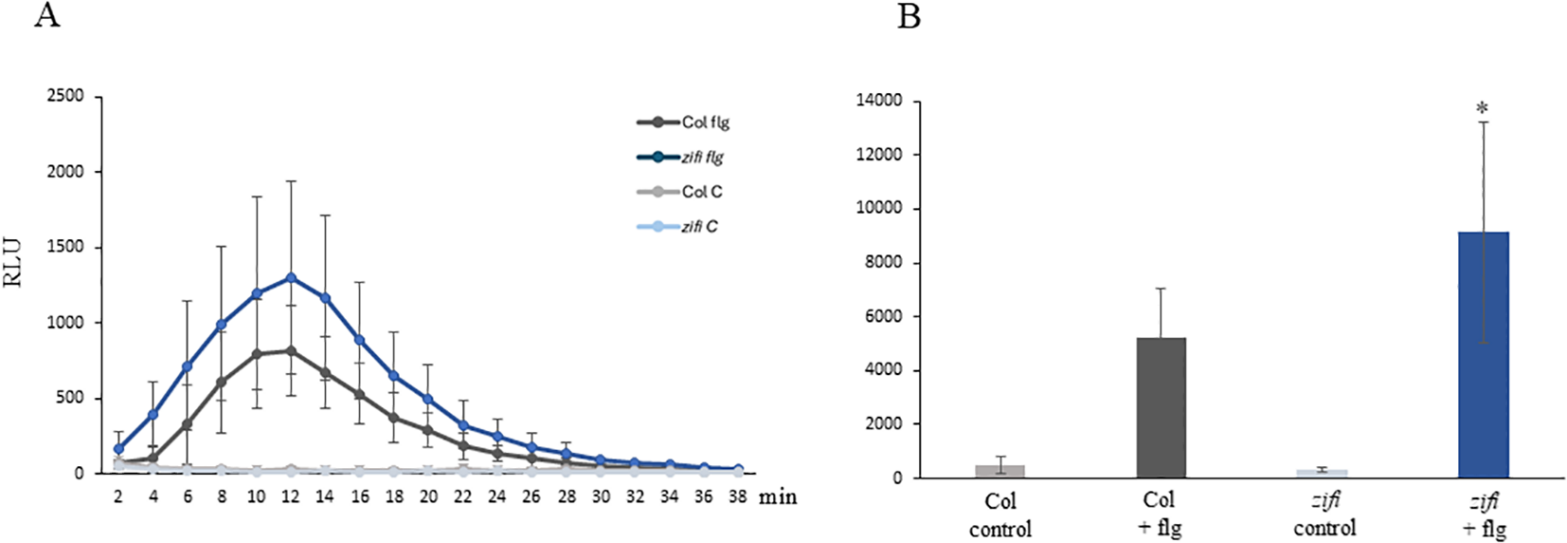
flg22 induced ROS production. Leaf discs of wild-type Col (gray) and *zifi1* (blue) were treated with water (lighter colors) or 100 nM of flg22 (darker colors). Shown are the mean relative light units of nine (Col flg22) to 10 (all other samples) biological replicates ± SD. **(A)** Relative light units at the time points indicated; **(B)** total intensity of relative light units from time point 2 to 38 min. Asterisks indicate significant differences between the wild-type Col and *zifi1* (*p < 0.05). The experiment was repeated three times with similar results.

As a test system for ROS production, leaf discs were treated with the bacterial elicitor peptide flg22. ROS levels increased after flg22 treatment with a maximum approximately 12 min after starting the measurement in both genotypes. However, the *zifi1* mutant showed significantly higher ROS production compared to the wild type (**Figure 6**).

**Figure 7:**
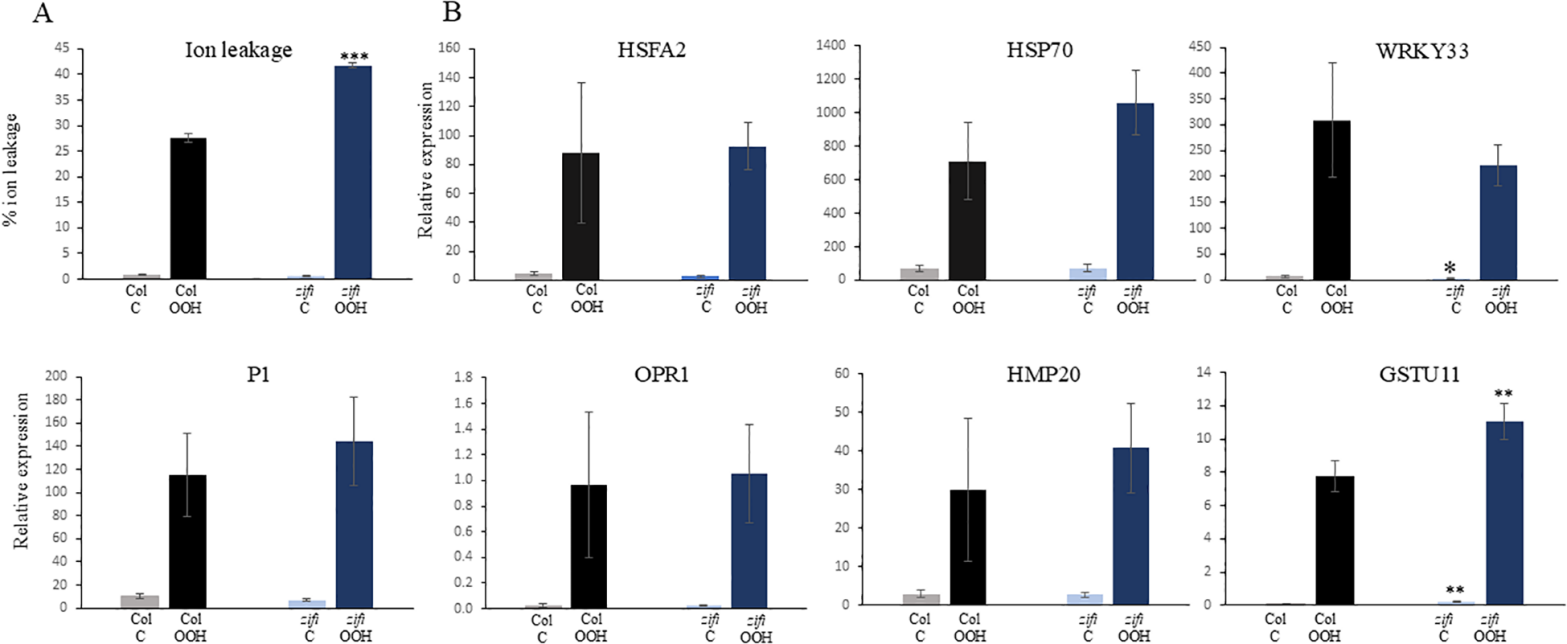
Response to oxidative stress in wild type (Col) and *zifi1*. Leaf discs of 6-week-old plants of wild type (gray/black bars) and *zifi1* (blue bars) were treated with 1 mM of tert-butyl hydroperoxide (OOH). Controls were treated with water. **(A)** Ion leakage after 6.5 h in percent of total ion leakage determined after killing cells by boiling. **(B)** Expression of oxidative stress-responsive genes after 1.5 h of OOH treatment. Shown are the means and standard deviations of four biological replicates. Asterisks indicate significant differences between the wild-type Col and *zifi1* (*p < 0.05; **p < 0.01; ***p < 0.001). The experiment was repeated four times with similar results.

BuOOH is an oxidative stress resulting in cell death. After 3 h of BuOOH addition, electrolyte leakage started to increase (**Supplementary Figure 6**). Ion leakage upon BuOOH treatment was significantly higher in *zifi1* and *nr1* 6.5 h after the start of the treatment compared to the wild type (**Figure 7A**; **Supplementary Figures 7**, **8**). This indicates that the ZIFI1 protein is involved in regulating the response to oxidative stress and a defect renders the mutant more sensitive to oxidative stress.

As explained above, several genes that are not or less induced by PGA in *zifi1* compared to the wild type are responsive to oxidative stress. This raises the question of whether there is also lower induction of these genes by oxidative stress. Expression of genes that show lower induction by PGA in *zifi1* compared to the wild type was analyzed 1.5 h after the start of BuOOH treatment. Expression of *HSFA2*, *P1*, and *HMP20* increased upon BuOOH treatment in Col. There was also induction by BuOOH in *zifi1*, and the expression levels were not lower compared to the wild type (**Figure 7B**). This indicates that ZIFI1 is not required for the general upregulation of these genes after BuOOH treatment.

## 4 Discussion

### 4.1 The CW-type zinc finger protein is involved in RES signaling and detoxification

While several components and mechanisms of the signaling pathway mediating the effects of JA-isoleucine have been described, less is known about RES-oxylipin signaling. This work identifies a CW-type zinc finger protein as a factor playing a role in RES-oxylipin signaling. Interestingly, ZIFI1 seems to be involved also in the transcriptional control of the response to an endogenous isothiocyanate and to a xenobiotic. This applies to a set of genes related to detoxification and *TolB-like*. A common property of RES-oxylipins, isothiocyanates, and several xenobiotics is reactivity, which might be important for the perception mechanism. The perception of RES-oxylipins is unclear. In Marchantia, OPDA is perceived by a COI1–JAZ receptor complex (Monte et al., 2022). So far, this seems not to be the case in *Arabidopsis*. As mentioned in the Introduction, the OPDA-binding protein cyclophilin 20-3 is only responsible for part of the responses. As an additional mechanism, covalent modification of proteins has been proposed which is supported by the finding that reactivity is crucial for biological activity (Almeras et al., 2003; Findling et al., 2018). Also, the result in this work that ZIFI1 is involved in the response to different RES supports that electrophile properties and the reaction with other molecules are involved in the RES signaling mechanism. In contrast to RES, the upregulation of ROS-responsive genes by BuOOH was not affected even though genes were tested that showed lower expression in *zifi1* upon PGA treatment. This indicates that ROS might signal through mechanisms different from RES-oxylipins and xenobiotics.

An important detoxification mechanism in plants and animals is the conjugation of RES with glutathione. Expression of GSTs is upregulated in response to many abiotic and biotic stresses (Wagner et al., 2002). GSTs have been shown to be important for the resistance of plants to herbicides and oxidative stress (Cummins et al., 1999; Ugalde et al., 2021). The increased sensitivity of *zifi1* to a xenobiotic and to oxidative stress is consistent with the lower expression of several GSTs in *zifi1*. In addition, the lower transcript levels of GSTs raised the question of whether the formation of glutathione–conjugates is impaired. Glutathione–conjugate levels after treatment with PGA were similar in the *zifi1* mutant and the wild type (**Supplementary Figure 9**). This result can be explained by the remaining GST expression and activity in the *zifi1* mutant. In addition, non-enzymatic conjugation might be responsible for glutathione–conjugate formation. Non-enzymatic conjugation has been shown to occur with OPDA and phytoprostanes (Dueckershoff et al., 2008).

In addition to detoxification, RES-oxylipins have also been implicated in the response to other stresses such as water deficit and hypoxia (see Introduction). Similarly, the gene ontology annotations of differentially expressed genes (**Table 1**) indicate the involvement of ZIFI1 in several stress responses such as water deficit and osmotic stress. This also suggests a connection of ZIFI1 signaling to the abscisic acid signaling pathway. Furthermore, the gene ontology annotations suggest that ZIFI1 participates in the transcriptional responses to bacteria and fungi. Therefore, it will be of interest to evaluate the relevance of ZIFI1 in the interaction with other organisms as well as in short-term responses and long-term adaptation to a variety of environmental factors.

### 4.2 Signaling by ZIFI1 might be connected to other transcription factors

The phenotypic differences of mutants with a defect in ZIFI1 compared to the wild type are not very strong. A possible reason might be functional redundancy with other transcription factors. The protein At1g02990 and the CW-type zinc finger protein At4g15730 are paralogs of ZIFI1 (**Supplementary Figure 10**) and might compensate for a defect in ZIFI1. In addition, the clade II TGA transcription factors TGA2, TGA5, and TGA6 are involved in regulating gene expression in response to RES-oxylipins and share functions with ZIFI1. The mutant *tga2,5,6* shows less gene induction upon treatment with OPDA, phytoprostanes, and PGA (Mueller et al., 2008; Stotz et al., 2013). Interestingly, TGA factors are in parallel involved in the SA, JA/ethylene, and RES-oxylipin signaling pathways. Consequently, several stress-related phenotypes of this mutant have been reported. The *tga2,5,6* mutant is impaired in SA-dependent systemic acquired resistance (Zhang et al., 2003). This mutant is more sensitive to the necrotrophic fungus *B. cinerea* (Zander et al., 2010) as well as to chemical stress by TIBA (Fode et al., 2008) or oxidative stress by BuOOH (Stotz et al., 2013). This poses an obvious similarity of phenotypes of the *tga2,5,6* triple mutant and the *zifi1* mutant. Both mutants are more sensitive to BuOOH and TIBA and induction by RES-oxylipins of genes related to detoxification is impaired. What is the relationship between TGA factors and ZIFI1? ZIFI1 and the TGA factors could work in parallel or hierarchically. Approximately one-third of the genes that are ZIFI1-dependently induced by PGA have been reported to be induced by A_1_-phytoprostanes dependent on TGA2,5,6, suggesting that signaling through ZIFI1 and TGA factors is connected. The gene expression data do not indicate altered expression of the *ZIFI1* gene in *tga2,5,6* or vice versa of the TGA factors in *zifi1*. This suggests a mutual effect on the activity of these transcription factors on a posttranscriptional level. For TGA factors, interactions with other proteins such as NPR1 and glutaredoxin480 have been reported which is important for regulating signaling activities (Zhang et al., 1999; Ndamukong et al., 2007). Further research is needed to decipher the connection between the TGA factors, the ZIFI1 protein, and the ZIFI1 paralogs in response to RES, ROS, and different stress factors.

### 4.3 What is the mechanism of ZIFI1 action and how is it regulated?

Zinc finger proteins constitute a large family of transcriptional regulators, and particularly, C2H2 zinc finger proteins have been reported to regulate the response to stress factors (Han et al., 2020). Less is known about the functions of the CW-type zinc finger protein subfamily. The CW-zinc finger domain comprises approximately 60 amino acids and contains conserved cysteine and tryptophane residues (at least four Cys and two Trp) (Perry and Zhao, 2003). CW domain proteins have been reported in vertebrates and higher plants but are present in diverse eukaryotes (**Supplementary Figure 11**). The homology of ZIFI1 to most of the CW domain proteins outside of the CW domain was rather low except for three CW domain proteins in rice (**Supplementary Figure 10**, see below). Regarding the function of proteins containing CW domains, some have been identified as readers of histone modifications (He et al., 2010; Hoppmann et al., 2011). Structural approaches have shown that the CW-type zinc finger proteins can recognize methylated lysine residues in histone H3. In *Arabidopsis*, this was shown for the well-studied CW domain protein SDG8/ASHH2 (Liu and Huang, 2018; Dobrovolska et al., 2020). In rice, three CW-type zinc finger proteins have been reported which bind histones depending on the methylation status. Mutants in one of the CW-type zinc finger protein genes are impaired in the development of reproductive structures (Zhang et al., 2017). Whether also the ZIFI1 protein described here regulates transcription by binding to DNA dependent on the status of histone modification needs further evaluation.

Another question arises regarding the regulation of the activity of the ZIFI1 protein. As shown above, the expression level of the *ZIFI1* gene is rather high (**Supplementary Figure 4**). In addition, there is only minor regulation of ZIFI1 on a transcriptional level in response to stresses (**Supplementary Figure 4C**). This suggests a posttranscriptional regulation. A common mechanism for regulating the activity of transcription factors is the interaction with other proteins. The sequence of ZIFI1 predicts a coiled coil domain at the C-terminus as a putative protein–protein interaction domain. It will be important to investigate interaction partners to elucidate whether the binding of other proteins is a regulation mechanism of ZIFI1 activity. Another possibility is a regulation by posttranslational modification of ZIFI1 such as phosphorylation. In addition, modification by RES and the redox state are possible mechanisms to regulate the activity of proteins (Dueckershoff et al., 2008; Stotz et al., 2013). Future research will reveal whether ZIFI1 is involved in regulating additional processes and how its activity is modulated.

## Data Availability

The mutant lines are available on request from the corresponding author. The transcriptome datasets for this study can be found in GEO, accession number: GSE286411 URL: https://www.ncbi.nlm.nih.gov/geo/query/acc.cgi?acc=GSE286411.

## References

[B1] Almeras E. Stolz S. Vollenweider S. Reymond P. Mene-Saffrane L. Farmer E. E. (2003). Reactive electrophile species activate defense gene expression in *Arabidopsis* . Plant J. 34, 205–216. doi: 10.1046/j.1365-313X.2003.01718.x 12694595

[B2] Burow M. Halkier B. A. (2017). How does a plant orchestrate defense in time and space? Using glucosinolates in Arabidopsis as case study. Curr. Opin. Plant Biol. 38, 142–147. doi: 10.1016/j.pbi.2017.04.009 28575680

[B3] Chen W. Singh K. B. (1999). The auxin, hydrogen peroxide and salicylic acid induced expression of the Arabidopsis GST6 promoter is mediated in part by an ocs element. Plant J. 19, 667–677. doi: 10.1046/j.1365-313x.1999.00560.x 10571852

[B4] Clough S. J. Bent A. F. (1998). Floral dip: a simplified method for Agrobacterium-mediated transformation of *Arabidopsis thaliana* . Plant J. 16, 735–743. doi: 10.1046/j.1365-313x.1998.00343.x 10069079

[B5] Croft K. P. C. Jüttner F. Slusarenko A. J. (1993). Volatile products of the lipoxygenase pathway evolved from *Phaseolus vulgaris* (L.) leaves inoculated with *Pseudomonas syringae* pv phaseolicola. Plant Physiol. 101, 13–24. doi: 10.1104/pp.101.1.13 12231661 PMC158642

[B6] Cummins I. Cole D. J. Edwards R. (1999). A role for glutathione transferases functioning as glutathione peroxidases in resistance to multiple herbicides in black-grass. Plant J. 18, 285–292. doi: 10.1046/j.1365-313X.1999.00452.x 10377994

[B7] Dobrovolska O. Brilkov M. Madeleine N. Odegard-Fougner O. Stromland O. Martin S. R.. (2020). The Arabidopsis (ASHH2) CW domain binds monomethylated K4 of the histone H3 tail through conformational selection. FEBS J. 287, 4458–4480. doi: 10.1111/febs.v287.20 32083791

[B8] Dueckershoff K. Mueller S. Mueller M. J. Reinders J. (2008). Impact of cyclopentenone-oxylipins on the proteome of *Arabidopsis thaliana.* Biochim. Biophys. Acta 1784, 1975–1985. doi: 10.1016/j.bbapap.2008.09.003 18848650

[B9] Farmer E. E. Mueller M. J. (2013). ROS-mediated lipid peroxidation and RES-activated signaling. Ann. Rev. Plant Biol. 64, 429–450. doi: 10.1146/annurev-arplant-050312-120132 23451784

[B10] Ferber E. Gerhards J. Sauer M. Krischke M. Dittrich M. T. Muller T.. (2020). Chemical priming by isothiocyanates protects against intoxication by products of the mustard oil bomb. Front. Plant Sci. 11, 887. doi: 10.3389/fpls.2020.00887 32676087 PMC7333730

[B11] Findling S. Stotz H. U. Zoeller M. Krischke M. Zander M. Gatz C.. (2018). TGA2 signaling in response to reactive electrophile species is not dependent on cysteine modification of TGA2. PloS One 13, e0195398. doi: 10.1371/journal.pone.0195398 29608605 PMC5880405

[B12] Fode B. Siemsen T. Thurow C. Weigel R. Gatz C. (2008). The Arabidopsis GRAS protein SCL14 interacts with class II TGA transcription factors and is essential for the activation of stress-inducible promoters. Plant Cell 20, 3122–3135. doi: 10.1105/tpc.108.058974 18984675 PMC2613660

[B13] Grun G. Berger S. Matthes D. Mueller M. J. (2007). Early accumulation of non-enzymatically synthesized oxylipins in *Arabidopsis thaliana* after infection with Pseudomonas syringae. Funct. Plant Biol. 34, 65–71. doi: 10.1071/FP06205 32689332

[B14] Han G. Lu C. Guo J. Qiao Z. Sui N. Qiu N.. (2020). C2H2 zinc finger proteins: master regulators of abiotic stress responses in plants. Front. Plant Sci. 11, 115. doi: 10.3389/fpls.2020.00115 32153617 PMC7044346

[B15] Hara M. Yatsuzuka Y. Tabata K. Kuboi T. (2010). Exogenously applied isothiocyanates enhance glutathione S-transferase expression in Arabidopsis but act as herbicides at higher concentrations. J. Plant Physiol. 167, 643–649. doi: 10.1016/j.jplph.2009.11.006 20031254

[B16] He F. Umehara T. Saito K. Harada T. Watanabe S. Yabuki T.. (2010). Structural insight into the zinc finger CW domain as a histone modification reader. Structure 18, 1127–1139. doi: 10.1016/j.str.2010.06.012 20826339

[B17] Hoppmann V. Thorstensen T. Kristiansen P. E. Veiseth S. V. Rahman M. A. Finne K.. (2011). The CW domain, a new histone recognition module in chromatin proteins. EMBO J. 30, 1939–1952. doi: 10.1038/emboj.2011.108 21522130 PMC3098480

[B18] Knieper M. Viehhauser A. Dietz K. J. (2023). Oxylipins and reactive carbonyls as regulators of the plant redox and reactive oxygen species network under stress. Antioxidants 12, 814–839. doi: 10.3390/antiox12040814 37107189 PMC10135161

[B19] Liu Y. Huang Y. (2018). Uncovering the mechanistic basis for specific recognition of monomethylated H3K4 by the CW domain of Arabidopsis histone methyltransferase SDG8. J. Biol. Chem. 293, 6470–6481. doi: 10.1074/jbc.RA117.001390 29496997 PMC5925821

[B20] Mittler R. Zandalinas S. I. Fichman Y. Van Breusegem F. (2022). Reactive oxygen species signalling in plant stress responses. Nat. Rev. Mol. Cell Biol. 23, 663–679. doi: 10.1038/s41580-022-00499-2 35760900

[B21] Monte I. Caballero J. Zamarreno A. M. Fernandez-Barbero G. Garcia-Mina J. M. Solano R. (2022). JAZ is essential for ligand specificity of the COI1/JAZ co-receptor. Proc. Natl. Acad. Sci. 119, e2212155119. doi: 10.1073/pnas.2212155119 36442090 PMC9894187

[B22] Montillet J. L. Leonhardt N. Mondy S. Tranchimand S. Rumeau D. Boudsocq M.. (2013). An abscisic acid-independent oxylipin pathway controls stomatal closure and immune defense in Arabidopsis. PloS Biol. 11, e1001513. doi: 10.1371/journal.pbio.1001513 23526882 PMC3602010

[B23] Mueller M. J. Berger S. (2009). Reactive electrophilic oxylipins: pattern recognition and signalling. Phytochemistry 70, 1511–1521. doi: 10.1016/j.phytochem.2009.05.018 19555983

[B24] Mueller S. Hilbert B. Dueckershoff K. Roitsch T. Krischke M. Mueller M. J.. (2008). General detoxification and stress responses are mediated by oxidized lipids through TGA transcription factors in Arabidopsis. Plant Cell 20, 768–785. doi: 10.1105/tpc.107.054809 18334669 PMC2329937

[B25] Ndamukong I. Abdallat A. A. Thurow C. Fode B. Zander M. Weigel R.. (2007). SA-inducible Arabidopsis glutaredoxin interacts with TGA factors and suppresses JA-responsive PDF1.2 transcription. Plant J. 50, 128–139. doi: 10.1111/j.1365-313X.2007.03039.x 17397508

[B26] Park S. W. Li W. Viehhauser A. He B. Kim S. Nilsson A. K.. (2013). Cyclophilin 20-3 relays a 12-oxo-phytodienoic acid signal during stress responsive regulation of cellular redox homeostasis. Proc. Natl. Acad. Sci. 110, 9559–9564. doi: 10.1073/pnas.1218872110 23671085 PMC3677464

[B27] Perry J. Zhao Y. (2003). The CW domain, a structural module shared amongst vertebrates, vertebrate-infecting parasites and higher plants. Trends Biochem. Sci. 28, 576–580. doi: 10.1016/j.tibs.2003.09.007 14607086

[B28] Raacke I. Mueller M. J. Berger S. (2006). Defects in allene oxide synthase and OPDA reductase alter the resistance to *P. syringae* and *B. cinerea* . J. Phytopathol. 154, 740–744. doi: 10.1111/j.1439-0434.2006.01191.x

[B29] Savchenko T. Kolla V. A. Wang C. Q. Nasafi Z. Hicks D. R. Phadungchob B.. (2014). Functional convergence of oxylipin and abscisic acid pathways controls stomatal closure in response to drought. Plant Physiol. 164, 1151–1160. doi: 10.1104/pp.113.234310 24429214 PMC3938610

[B30] Schneeberger K. Ossowski S. Lanz C. Juul T. Petersen A. H. Nielsen K. L.. (2009). SHOREmap: simultaneous mapping and mutation identification by deep sequencing. Nat. Methods 6, 550–551. doi: 10.1038/nmeth0809-550 19644454

[B31] Stotz H. U. Mueller S. Zoeller M. Mueller M. J. Berger S. (2013). TGA transcription factors and jasmonate-independent COI1 signalling regulate specific plant responses to reactive oxylipins. J. Exp. Bot. 64, 963–975. doi: 10.1093/jxb/ers389 23349138 PMC3580818

[B32] Szyroki A. Ivashikina N. Dietrich P. Roelfsema M. R. Ache P. Reintanz B.. (2001). KAT1 is not essential for stomatal opening. Proc. Natl. Acad. Sci. U.S.A. 98, 2917–2921. doi: 10.1073/pnas.051616698 11226341 PMC30240

[B33] Ugalde J. M. Lamig L. Herrera-Vasquez A. Fuchs P. Homagk M. Kopriva S.. (2021). A dual role for glutathione transferase U7 in plant growth and protection from methyl viologen-induced oxidative stress. Plant Physiol. 187, 2451–2468. doi: 10.1093/plphys/kiab444 34599589 PMC8644736

[B34] Wagner U. Edwards R. Dixon D. P. Mauch F. (2002). Probing the diversity of the Arabidopsis glutathione S-transferase gene family. Plant Mol. Biol. 49, 515–532. doi: 10.1023/A:1015557300450 12090627

[B35] Yamauchi Y. Kunishima M. Mizutani M. Sugimoto Y. (2015). Reactive short-chain leaf volatiles act as powerful inducers of abiotic stress-related gene expression. Sci. Rep. 5, 8030. doi: 10.1038/srep08030 25619826 PMC4306126

[B36] Zander M. La Camera S. Lamotte O. Metraux J. P. Gatz C. (2010). *Arabidopsis thaliana* class-II TGA transcription factors are essential activators of jasmonic acid/ethylene-induced defense responses. Plant J. 61, 200–210. doi: 10.1111/j.1365-313X.2009.04044.x 19832945

[B37] Zhang Y. Fan W. Kinkema M. Li X. Dong X. (1999). Interaction of NPR1 with basic leucine zipper protein transcription factors that bind sequences required for salicylic acid induction of the *PR-1* gene. Proc. Natl. Acad. Sci. 96, 6523–6528. doi: 10.1073/pnas.96.11.6523 10339621 PMC26915

[B38] Zhang Y. Tessaro M. J. Lassner M. Li X. (2003). Knockout analysis of Arabidopsis transcription factors TGA2, TGA5, and TGA6 reveals their redundant and essential roles in systemic acquired resistance. Plant Cell 15, 2647–2653. doi: 10.1105/tpc.014894 14576289 PMC280568

[B39] Zhang Z. Zhang F. Cheng Z. J. Liu L. L. Lin Q. B. Wu F. Q.. (2017). Functional characterization of rice CW-domain containing zinc finger proteins involved in histone recognition. Plant Sci. 263, 168–176. doi: 10.1016/j.plantsci.2017.06.013 28818372

[B40] Zimmermann P. Hirsch-Hoffmann M. Hennig L. Gruissem W. (2004). GENEVESTIGATOR. Arabidopsis microarray database and analysis toolbox. Plant Physiol. 136, 2621–2632. doi: 10.1104/pp.104.046367 15375207 PMC523327

