# Supplementary material for: A CW-type zinc finger protein is involved in RES-oxylipin signaling and the response to abiotic stress in Arabidopsis thaliana
Source: Front Plant Sci. 2025 Mar 12;16:1535643. doi: 10.3389/fpls.2025.1535643 (PMC11937836; doi:10.3389/fpls.2025.1535643)
Supplement: Supplementary file 1 [file DataSheet1.pdf]

Experimental design:

Screening of the mutagenized GST6::LUC line for RES-oxylipin signaling mutants

↓

Identification of the mutant *non-responsive 1* (*nr1*) with lower luciferase activity upon PGA treatment

↓

Backcross of *nr1* to the GST6::LUC line,
Generation of the F2 population by self–pollination of F1 plants → determination of recessive inheritance of mutant phenotype (lower luciferase activity upon PGA treatment in the F2 population)

↓

Selection of 50 plants in the F2 generation with mutant phenotype (lower luciferase activity upon PGA treatment)

↓

Next Generation Mapping,
Identification of the defect in the *ZIFI1* gene (At3g62900) as the most probable responsible mutation for the *nr1* phenotype

↓

Confirmation using a T-DNA insertion line (termed *zifi1*) with a defect in the *ZIFI1* gene (At3g62900)

↓

Analyses of transcriptomes and stress responses in *nr1* and *zifi1* mutants

lines used:

Col: wild type ecotype Columbia, Col-0

GST6::LUC: transgenic line expressing luciferase under the control of the *GST6* promoter, used for mutagenesis and as a control line for the *nr1* mutant

nr1: mutant selected by screening for plants with lower luciferase activity upon PGA treatment, contains a point mutation in the *ZIFI1* gene on chromosome 3, position 23252228, background line is the GST6::LUC line

zifi1: mutant containing a T-DNA insertion in the *ZIFI1* gene At3g62900, line SALK_064820, the corresponding wild type is Col-0

F-box: mutant containing a T-DNA insertion in the *F-box* gene At3g61340, line SALK_130710

Suppl. Fig. 1 explaining the experimental design and the lines used

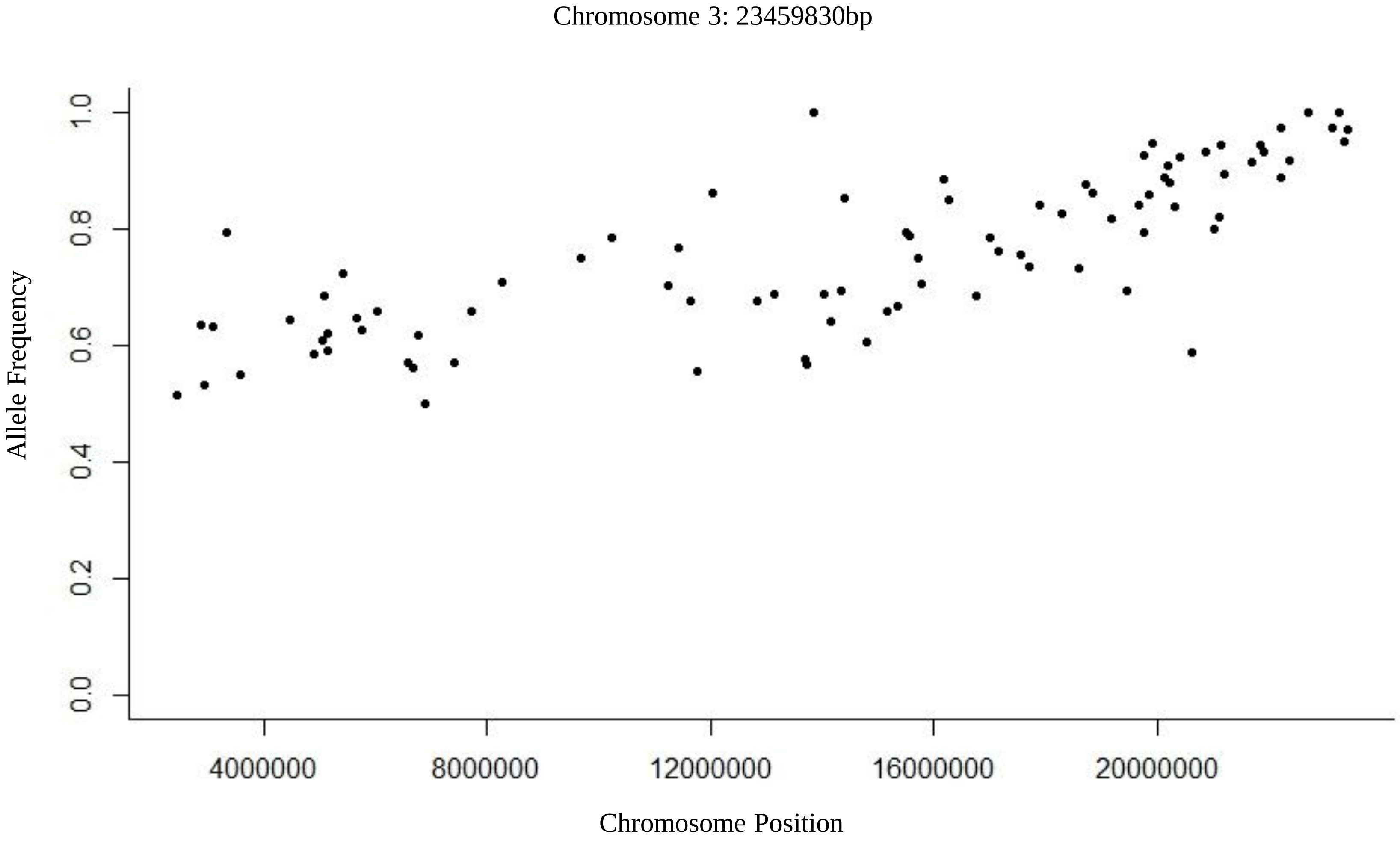

Suppl. Fig. 2: Allele frequency of polymorphisms on chromosome 3. The figure was generated with SHOREmap v3.5. In total, 120 SNPs, 62 insertions and 32 deletions were detected in *nr1* compared to the control line. Chromosome 3 shows a region with high allele frequency which was not observed on the other chromosomes.

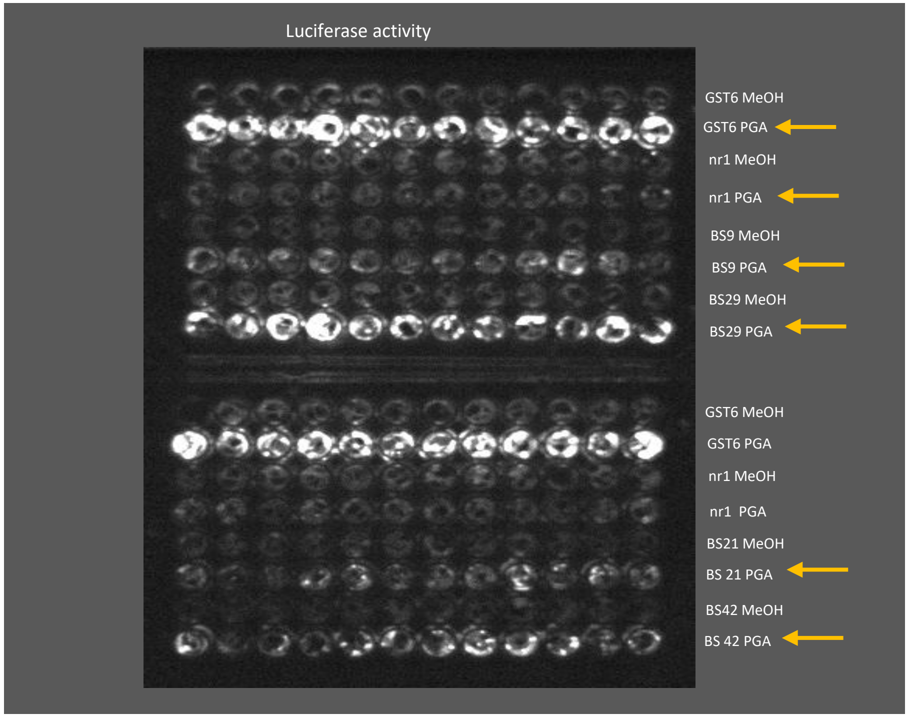

Suppl. Fig. 3: complementation of the *nr1* phenotype by expression of the wild type *ZIFI1* gene. Luciferase activity of lines expressing the WT-*ZIFI1* gene in the *nr1* background under control of the 35S promoter. 10 d old seedlings were treated with mock or 75 µM PGA. Luciferase activity was determined after 6 h. shown are 12 replicates of each genotype and treatment. The experiment was repeated two times with similar results.

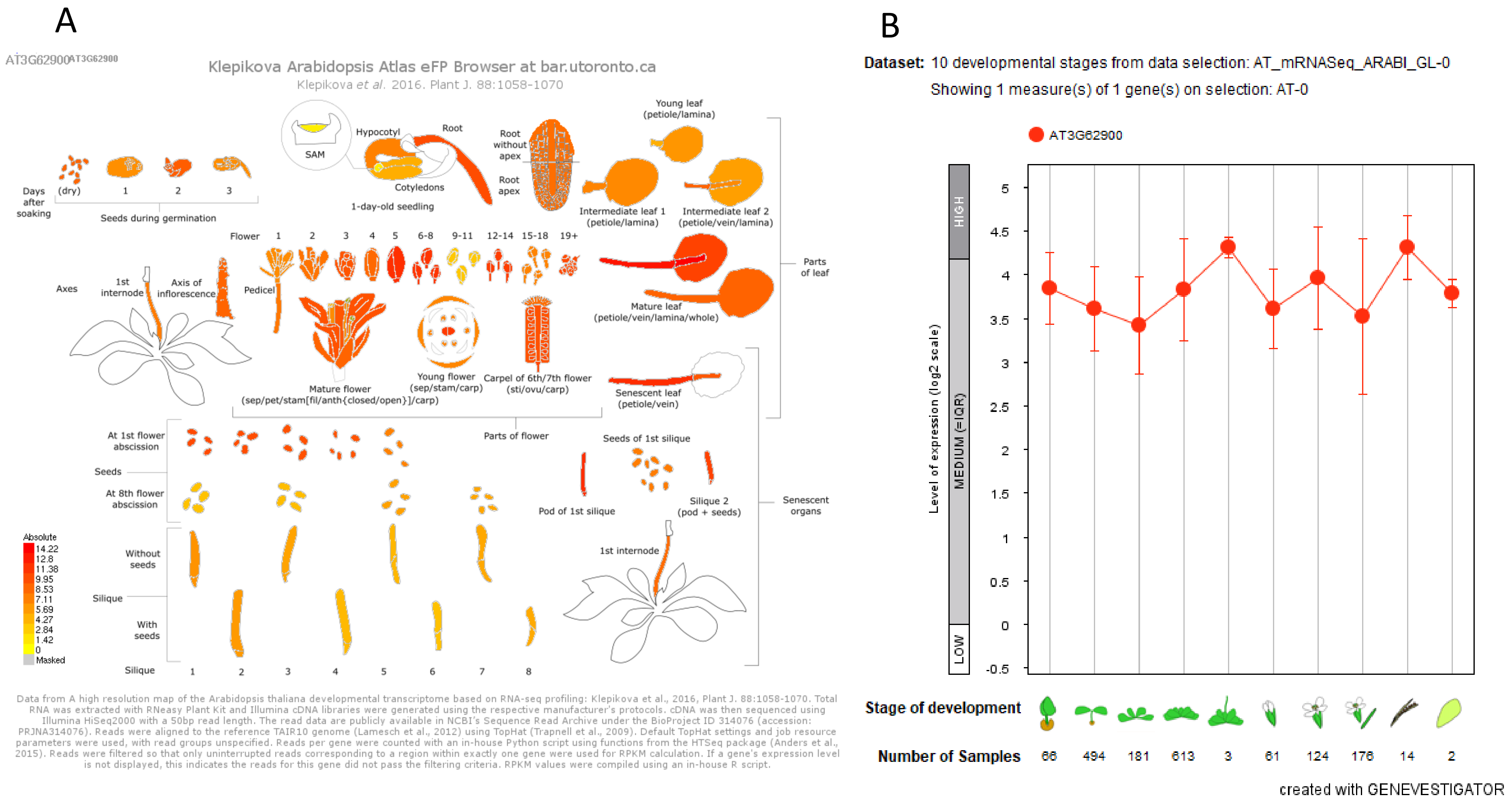

Suppl. Fig. 4: Expression of *ZIFI1* (At3g62900). A: expression of At3g62900 in different tissues and developmental stages according to the eFP Browser (Klepikova et al. 2016). B: expression of At3g62900 in developmental stages according to genevestigator (Zimmermann et al. 2004). C: alteration of expression of At3g62900 by perturbation with filter fold change >2 and p-value < 0.05 according to genevestigator (Zimmermann et al. 2004; created 8 August 2023)

Suppl. Fig. 4C

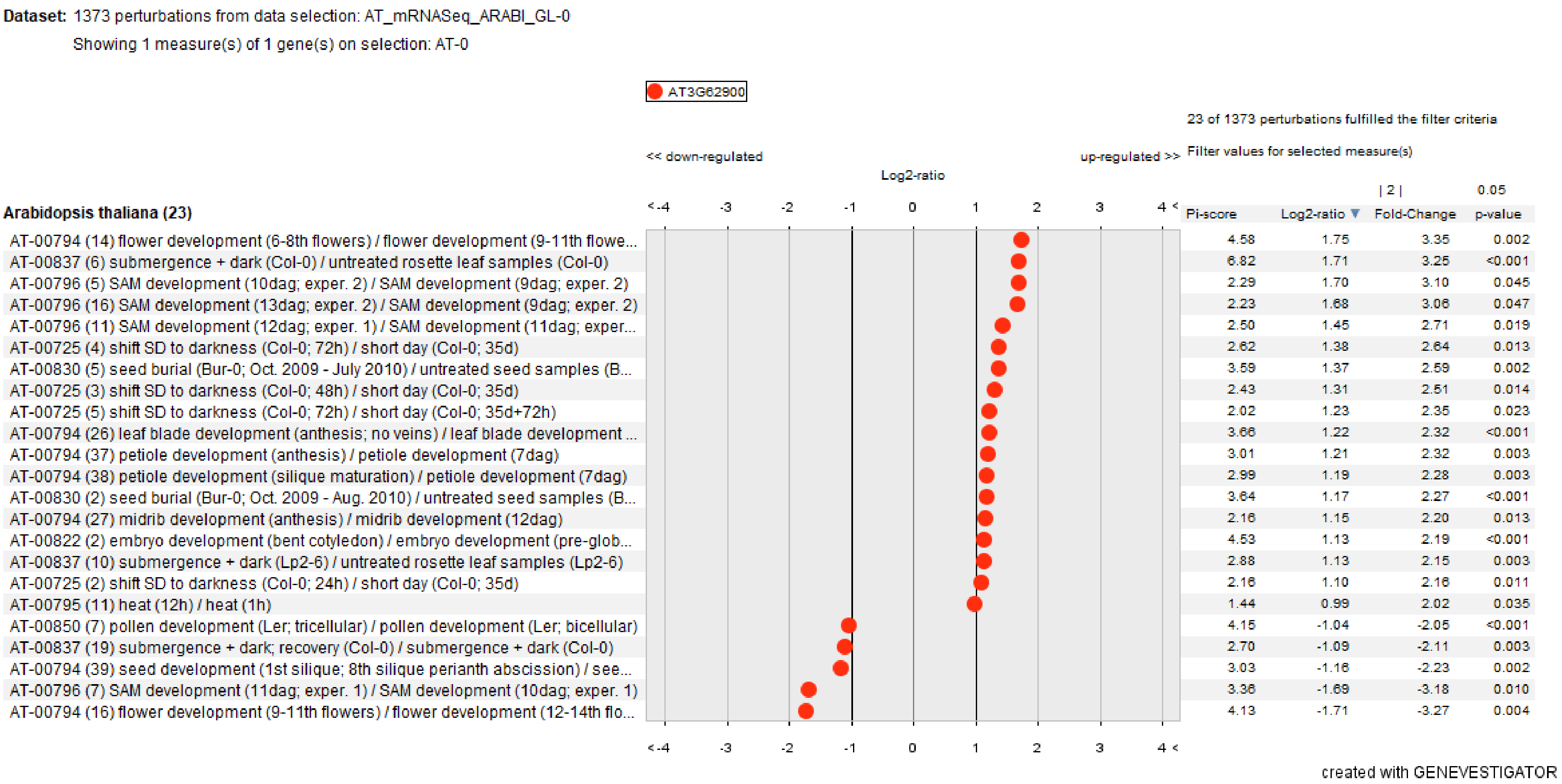

Suppl. Fig. 4: Expression of *ZIFI1* (At3g62900). A: expression of At3g62900 in different tissues and developmental stages according to the eFP Browser (Klepikova et al. 2016). B: expression of At3g62900 in developmental stages according to genevestigator (Zimmermann et al. 2004). C: alteration of expression of At3g62900 by perturbation with filter fold change >2 and p-value < 0.05 according to genevestigator (Zimmermann et al. 2004; created 8 August 2023)

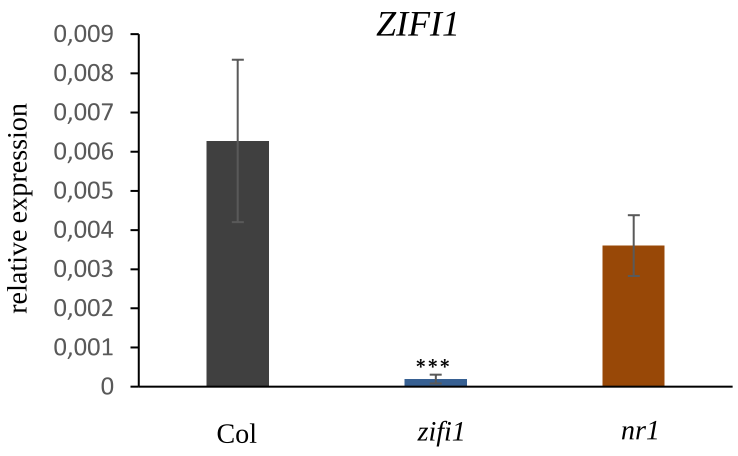

Suppl. Fig. 5: Expression of *ZIFI1* in different mutant lines. B: Seedlings of wild type Col (grey bars), *nr1* (green bars) and the T-DNA-insertion line *zifi1* (blue bars) were treated with 75 µM PGA for 4 h. Expression is relative to *SAND*. Shown is the mean of six biological replicates $\pm$ sd. Asterisks indicate significant differences between the wild type Col and mutant lines (* $p < 0.05$; *** $p < 0.001$). The experiment was repeated with similar results.

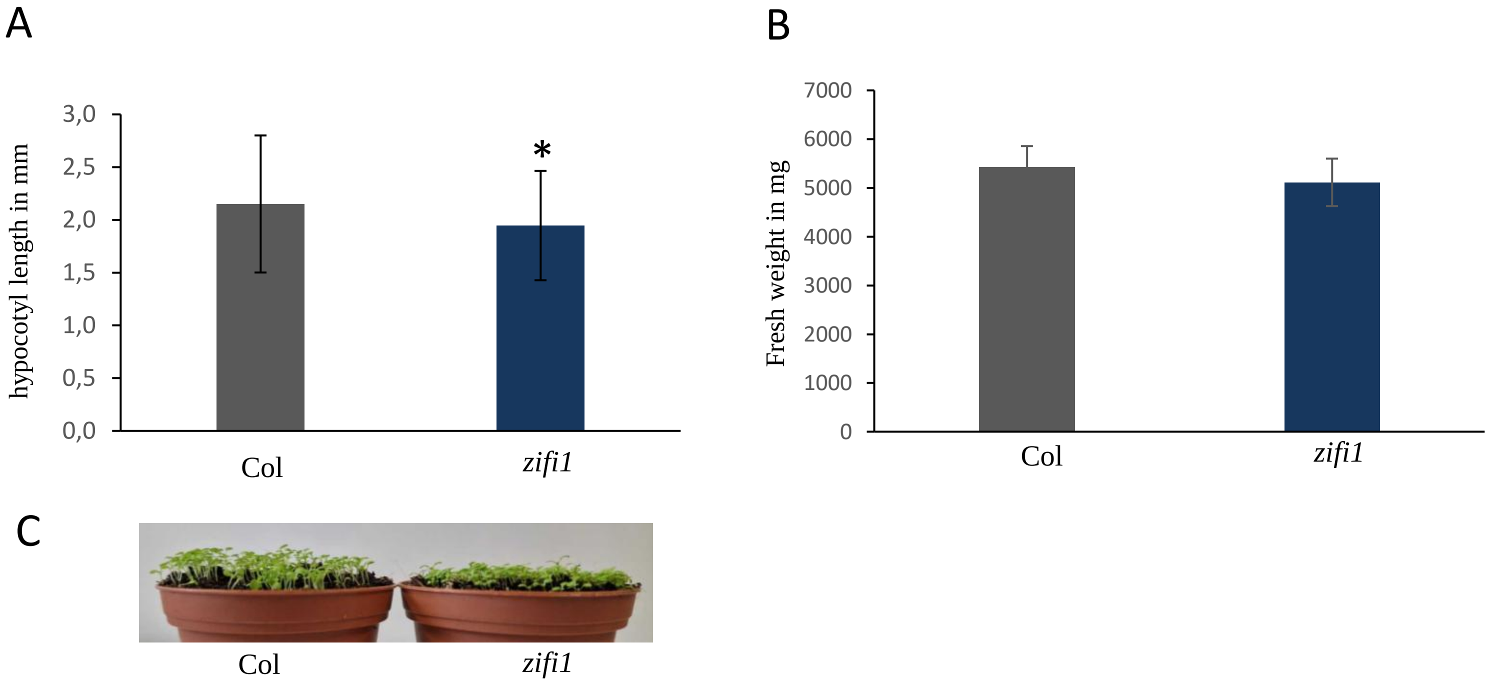

Suppl. Fig. 6: growth phenotypes of *zifi1*. A: hypocotyl length of 7 d old seedlings of wild type Col (grey bars) and the T-DNA-insertion line *zifi1* (blue bars) Shown is the mean of 104 (Col) and 96 (*zifi1*) seedlings $\pm$ sd. Asterisks indicate significant differences between the wild type Col and mutant lines (* $p < 0.05$). The experiment was repeated several times, a significant difference was observed in three of six experiments.
B: fresh weight of six week old soil grown plants of wild type Col (grey bars) and the T-DNA-insertion line *zifi1* (blue bars). Shown is the mean of 8 plants $\pm$ sd. The experiment was repeated four times, a significant difference was observed in two experiments.
C: photo of 12 d old seedlings grown in short day conditions in soil.

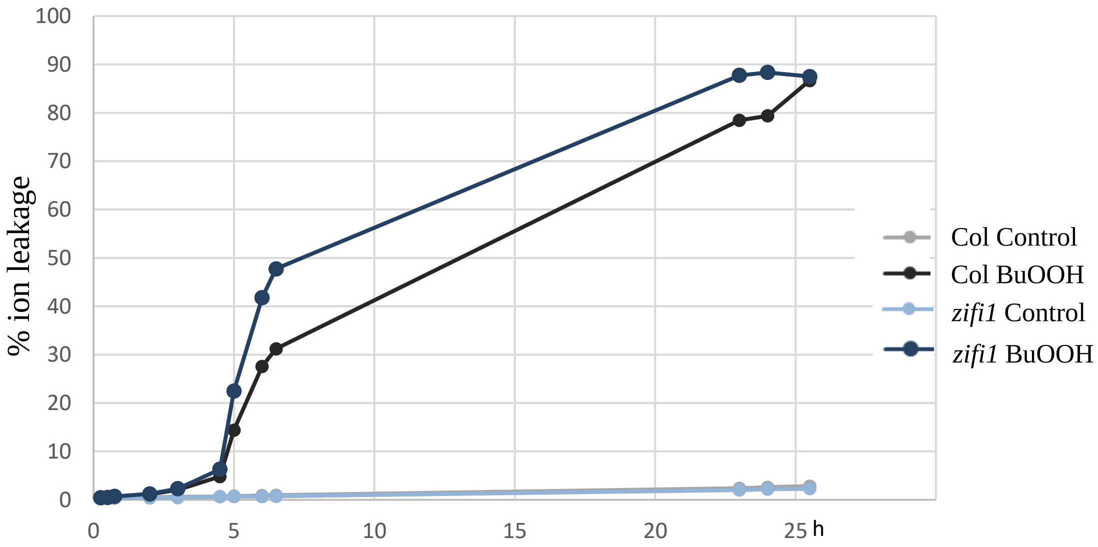

Suppl. Fig. 7. Cell death in wild type (Col) and *zifi1*. Leaf discs of 6 week old plants of wild type (grey/black lines) and *zifi1* (blue lines) were treated with 1 mM tert-butylhydroperoxide (BuOOH) and ion leakage was measured. Total (100%) ion leakage was determined after killing cells by boiling. Controls were treated with water. Shown are the means of four biological replicates. The experiment was repeated four times with similar results.

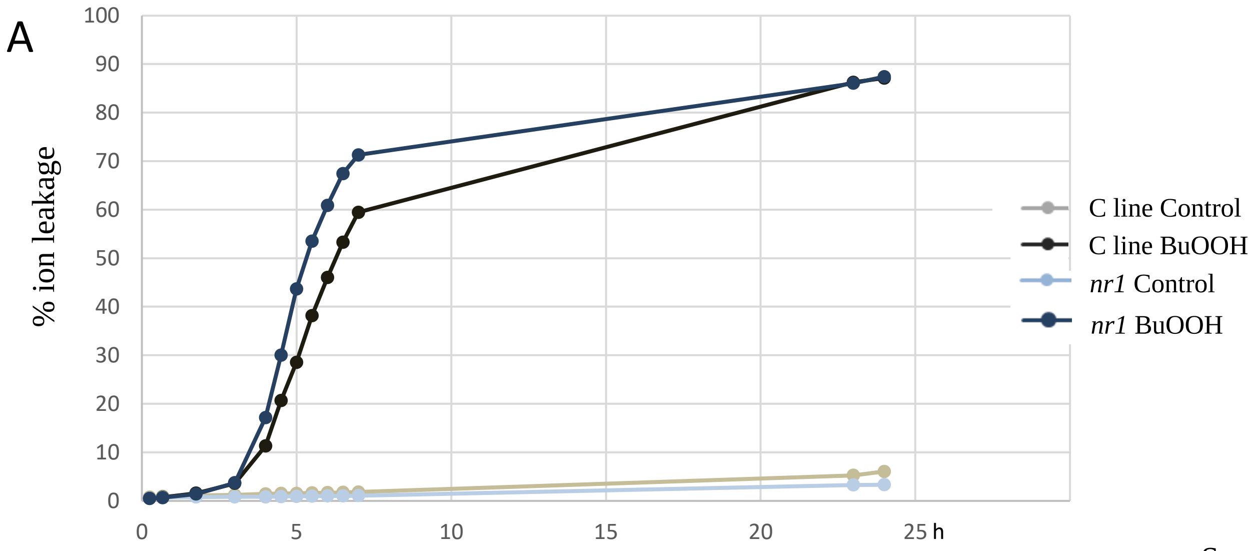

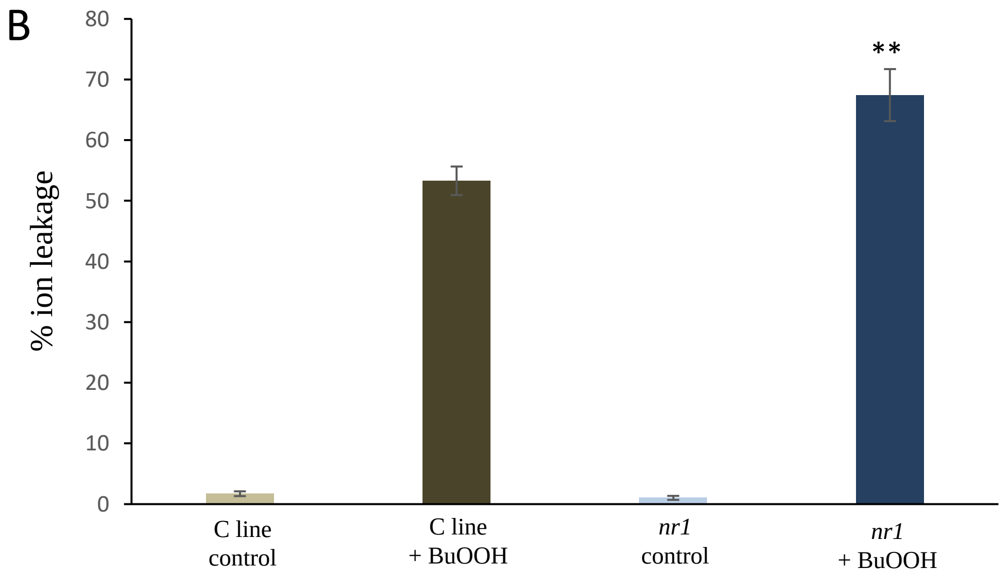

Suppl. Fig. 8. Cell death in control line and *nr1*. Leaf discs of 6 week old plants control line (grey/black lines/bars) and *nr1* (blue lines/bars) were treated with 1 mM tert-butylhydroperoxide (BuOOH) and ion leakage was measured. Total (100%) ion leakage was determined after killing cells by boiling. Controls were treated with water. Shown are the means of four biological replicates. The experiment was repeated four times with similar results. A: time course B: ion leakage after 6.5 h. Asterisks indicate significant differences between the wild type Col and mutant lines (** $p < 0.01$).

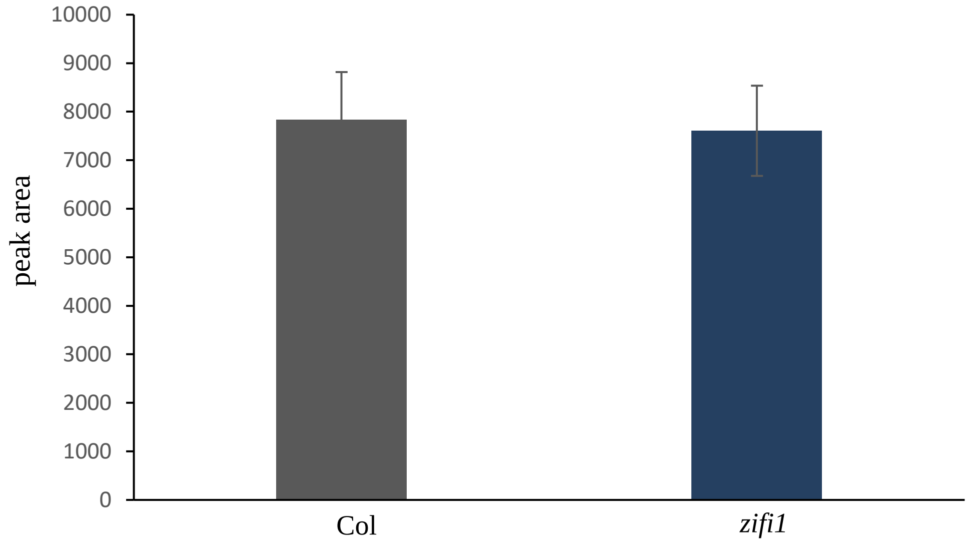

Suppl. Fig. 9: levels of PGA-glutathione conjugate. Seedlings of wild type Col (grey bars) and *zifi1* (blue bars) were treated with mock or 75 µM PGA for 24 h. Shown is the mean of the peak area with m/z of 642.307 Da of six biological replicates <u>+</u> sd. The experiment was repeated with similar results. No PGA-glutathione conjugate was detectable in mock treated seedlings.

A

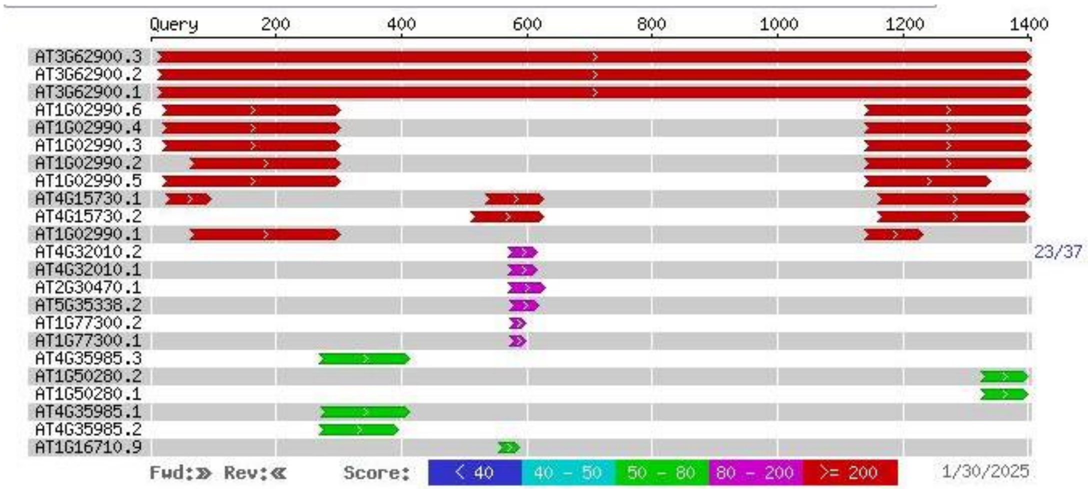

B

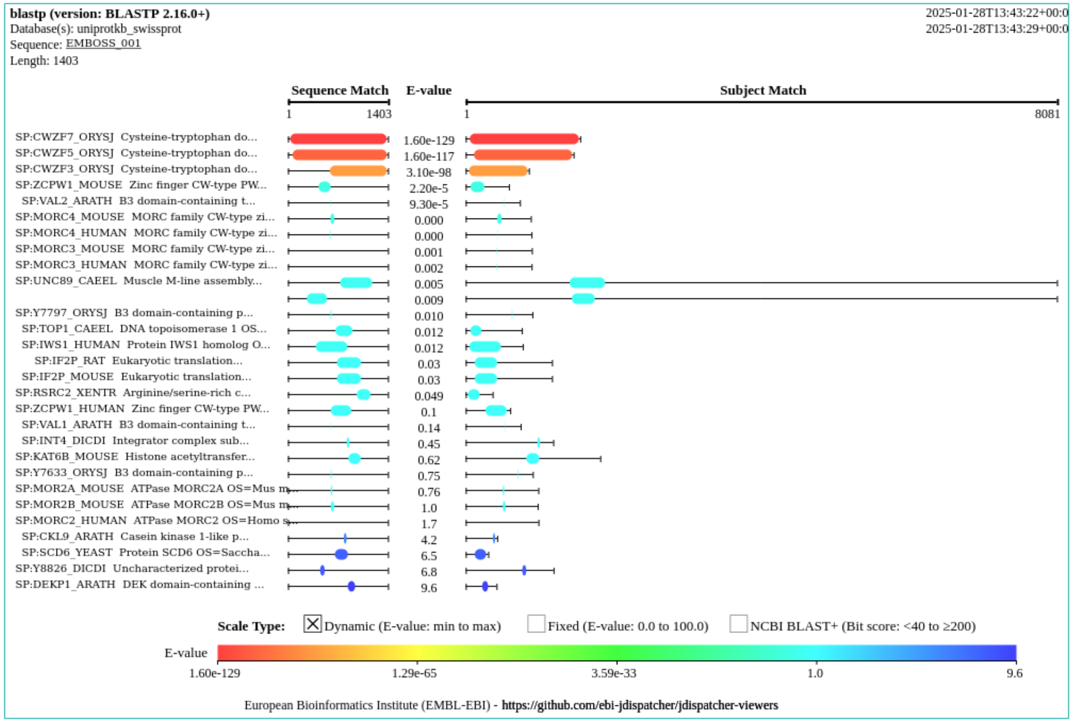

Suppl. Fig. 10: ZIFI1 paralogs and orthologs
(A): Results of a BLAST search with ZIFI1 protein sequence (At3g62900) in the Araport11 protein database
(B): Results of a BLAST search with ZIFI1 protein sequence (At3g62900) in the NCBI protein database, created with: https://github.com/ebi-jdispatcher-viewers

Altschul, S.F., Madden, T.L., Schäffer, A.A., Zhang, J., Zhang Z., Miller, W., Lipman, D.J. (1997) Gapped BLAST and PSI-BLAST: a new generation of protein database search programs. *Nucleic Acids Res*., 25, 3389-3402.

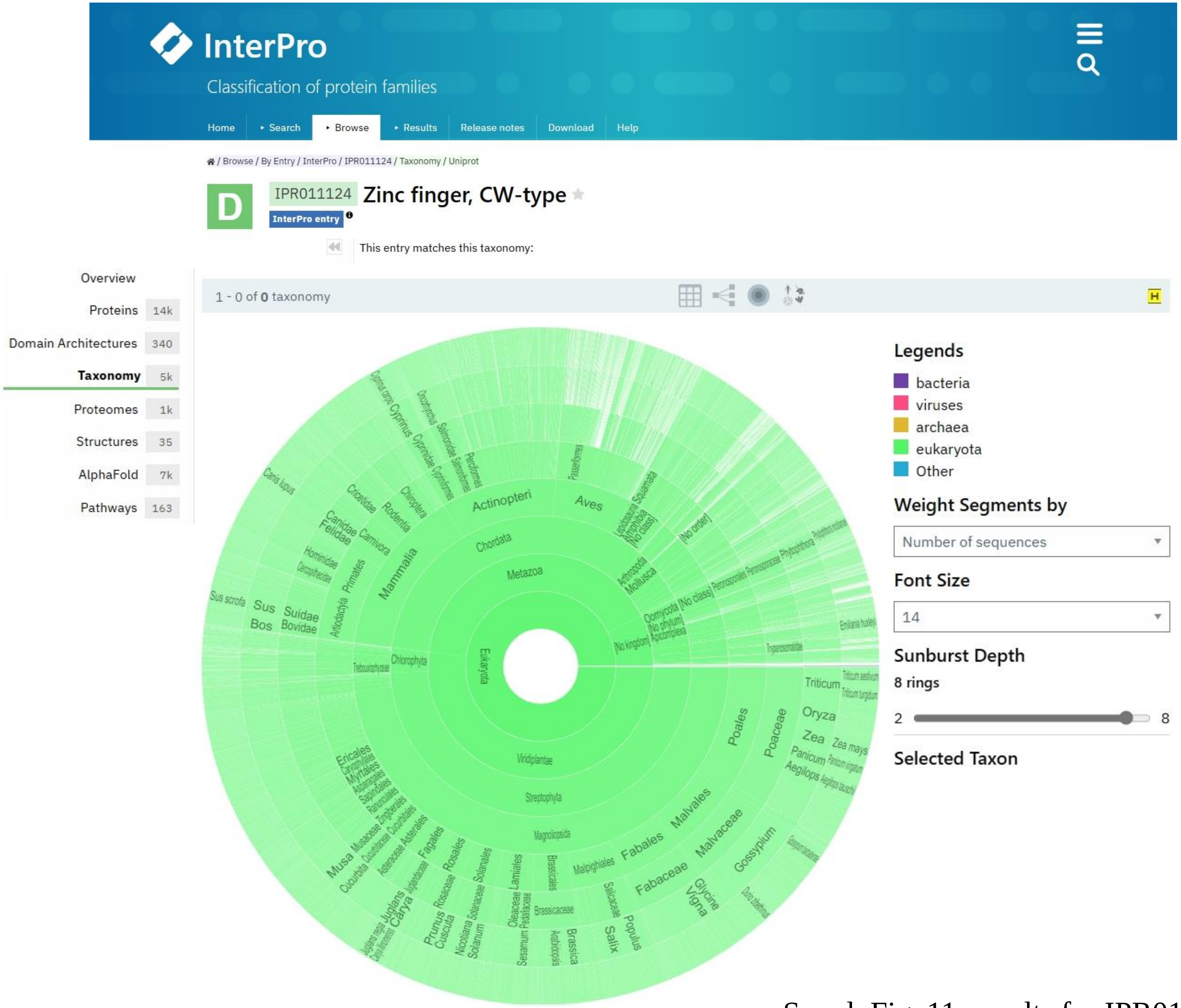

Suppl. Fig. 11: results for IPR011124 with the taxonomy-tool of InterPro, EMBL-EBI, UK
https://www.ebi.ac.uk/interpro/entry/InterPro/IPR011124/
